# Oncolytic strategy using new bifunctional HDACs/BRD4 inhibitors against virus-associated lymphomas

**DOI:** 10.1371/journal.ppat.1011089

**Published:** 2023-01-13

**Authors:** Jungang Chen, Zhengyu Wang, Tran Phuc, Zhigang Xu, Donglin Yang, Zhengzhu Chen, Zhen Lin, Samantha Kendrick, Lu Dai, Hong-yu Li, Zhiqiang Qin

**Affiliations:** 1 Department of Pathology, Winthrop P. Rockefeller Cancer Institute, University of Arkansas for Medical Sciences, Little Rock, Arkansas, United States of America; 2 Department of Pharmaceutical Sciences, College of Pharmacy, University of Arkansas for Medical Sciences, Little Rock, Arkansas, United States of America; 3 College of Pharmacy, Chongqing University of Arts and Sciences, Yongchuan, Chongqing, China; 4 Department of Pathology, Tulane University Health Sciences Center, Tulane Cancer Center, New Orleans, Louisiana, United States of America; 5 Department of Biochemistry and Molecular Biology, University of Arkansas for Medical Sciences, Little Rock, Arkansas, United States of America; University of Washington, UNITED STATES

## Abstract

Primary effusion lymphoma (PEL) caused by Kaposi sarcoma–associated herpesvirus (KSHV) is an aggressive malignancy with poor prognosis even under chemotherapy. Currently, there is no specific treatment for PEL therefore requiring new therapies. Both histone deacetylases (HDACs) and bromodomain-containing protein 4 (BRD4) have been found as therapeutic targets for PEL through inducing viral lytic reactivation. However, the strategy of dual targeting with one agent and potential synergistic effects have never been explored. In the current study, we first demonstrated the synergistic effect of concurrently targeting HDACs and BRD4 on KSHV reactivation by using SAHA or entinostat (HDACs inhibitors) and (+)-JQ1 (BRD4 inhibitor), which indicated dual blockage of HDACs/BRD4 is a viable therapeutic approach. We were then able to rationally design and synthesize a series of new small-molecule inhibitors targeting HDACs and BRD4 with a balanced activity profile by generating a hybrid of the key binding motifs between (+)-JQ1 and entinostat or SAHA. Upon two iterative screenings of optimized compounds, a pair of epimers, 009P1 and 009P2, were identified to better inhibit the growth of KSHV positive lymphomas compared to (+)-JQ1 or SAHA alone at low nanomolar concentrations, but not KSHV negative control cells or normal cells. Mechanistic studies of 009P1 and 009P2 demonstrated significantly enhanced viral reactivation, cell cycle arrest and apoptosis in KSHV+ lymphomas through dually targeting HDACs and BRD4 signaling activities. Importantly, *in vivo* preclinical studies showed that 009P1 and 009P2 dramatically suppressed KSHV+ lymphoma progression with oral bioavailability and minimal visible toxicity. These data together provide a novel strategy for the development of agents for inducing lytic activation-based therapies against these viruses-associated malignancies.

## Author summary

Oncolytic strategy through inducing viral lytic reactivation represents a promising direction against oncogenic herpesvirus associated malignancies. Although histone deacetylases (HDACs) and bromodomain-containing protein 4 (BRD4) have been reported to regulate Kaposi sarcoma–associated herpesvirus (KSHV) lytic reactivation, the potential synergistic effects by dual targeting (including the development of bifunctional HDACs/BRD4 inhibitors) have never been investigated. Here we for the first time showed the synergistic effect on KSHV reactivation with the combination of HDACs and BRD4 inhibitors. Based on that, we designed and synthesized a series of new small-molecule compounds dually targeting HDACs and BRD4 with a balanced activity profile through chemical modification of current HDACs and BRD4 inhibitors. After screenings with KSHV+ lymphoma systems, we identified 009P1 and 009P2, a pair of epimers, displaying better anticancer activities for KSHV+ lymphomas than current HDACs or BRD4 inhibitors even at low nanomolar concentrations, and with no visible toxicity on normal cells. Our further mechanistic studies showed that both new compounds significantly enhanced viral lytic genes expression through dually targeting HDACs and BRD4 signaling, resulting in KSHV+ lymphomas cell cycle arrest and apoptosis. In addition, the transcriptomic analysis identified cellular profile altered by these new compounds in PEL cells. By using an established PEL xenograft model, we showed that both 009P1 and 009P2 dramatically suppressed KSHV+ lymphoma progression in mice via either i.p. or oral delivery. Taken together, our study demonstrate that these new HDACs/BRD4 dual inhibitors may represent promising agents against virus-associated malignancies.

## Introduction

Kaposi’s sarcoma associated herpesvirus (KSHV), an oncogenic herpesvirus, is associated with the development of Kaposi’s sarcoma (KS), multicentric Castleman’s disease (MCD) and several lymphomas including primary effusion lymphoma (PEL) [1]. KSHV associated PEL, also known as body cavity-based lymphoma, is a classical neoplasm involving cells of the B cell lineage, of which the characteristic presents as an effusion with on detectable tumor in individuals and with poor prognosis [2]. Current treatment options primarily depend on chemotherapy, immunotherapy, and antiviral therapy, however, these therapies are often ineffective due to drug resistance and dose-limiting toxicities [3], therefore, the development of more effective agents with improved safety windows are urgently needed.

Similar to other human herpesviruses, KSHV have two alternative life cycle programs, the latent and lytic phases, which are essential for the maintenance of viral infections and tumorigenesis [4]. Generally, the latency phase is the main contributor of persistence in virus infected tumor cells where only a small subset of viral latent genes are expressed [4]. In contrast, the lytic phase is characterized by the expression of almost all of the viral encoded genes, replication of genomic DNA and ultimately release of mature virions leading to destruction of host cells [5]. Due to the limited efficacy of currently available antivirals to latently infected cells, increasing studies have sought to investigate the feasibility of an oncolytic strategy via reactivating endogenous latent viruses with chemical agents as an attractive therapy to target latent infection while simultaneously inducing cell death to eradicate virally infected reservoirs [6,7]. Recently, several groups identified oncolytic strategies that inducing lytic reactivation of KSHV and Epstein-Barr virus (EBV) as a promising approach for their-associated malignancies [8,9]. Both histone deacetylases (HDACs) and bromodomain-containing protein 4 (BRD4) are validated as therapeutic targets in KSHV associated lymphomas, strongly supported by the excellent anticancer activity of specific inhibitors for these specialized lymphomas *in vitro* and *in vivo* [10,11]. For example, suberoylanilide hydroxamic acid (Vorinostat, SAHA, a HDAC inhibitor) and (+)-JQ1 (a BRD4 inhibitor) display an impressive efficacy against KSHV-associated lymphomas in cultured cells, animal models, and patients [10,12,13]. However, acquired single-drug resistance [14,15], relative narrow therapeutic indexes [16], and poor pharmacokinetic profiles [17], such as short elimination half-life and low oral bioavailability, limit SAHA and (+)-JQ1 therapeutic potentials.

HDACs are a group of enzymes that remove acetyl groups from ε-N-acetyl lysine amino acids in histones and other proteins, such as tubulin, and play an important role in the epigenetic control of cellular gene transcription [18]. Interestingly, studies found that HDACs inhibition are required for KSHV reactivation and treatment of PEL cells with pan or specific isoform HDAC inhibitors, such as valproic acid, sodium butyrate (NaB), SAHA and suberoyl bis-hydroxamic acid (SBHA), effectively induces KSHV reactivation [11,19,20]. Current reports found several inhibitors, such as SBHA and SAHA, have anti-PEL ability, of which the mechanisms were partly associated with viral reactivation [10,11]. And, the previous data found that SAHA in combination with Bortezomib, a potent activator of KSHV/EBV lytic cycle, synergistically eradicates PEL cells without leading to increased viremia [10,21], as well as synergistically suppresses the growth of EBV-associated lymphomas and other cancers in murine models [22].

BRD4, a member of bromodomain and extra-terminal protein (EBT) family, plays a pivotal role in gene transcription and cancer development. As a reader of the histone code, the mechanism of BRD4 regulation of gene transcription has been implicated as interacting with the transcription elongation factor P-TEFb and RNA polymerase II through accumulating on the hyper-acetylated histone regions along the chromatin [23]. Earlier work verified the highly sensitivity of PEL cells to BET inhibitors and silencing of BRD4 effectively blocked cell proliferation and cell-cycle progression by the inhibition of c-Myc, whose stability is regulated by BRD4 [24]. Subsequent studies identified (+)-JQ1 as an epigenetic activator of KSHV lytic reactivation, of which the mechanisms might be associated with disrupting Rad21-dependent conformational control of the latency [25] and even disrupting the *MYC* superenhancer function [26]. Interestingly, several studies demonstrated anti-PEL activity of BRD4 inhibitors *in vitro* and *in vivo* [27]. Moreover, JQ1 in combination with PEP005, an inducer for KSHV reactivation, synergistically inhibited PEL growth *in vitro* and *in vivo* in the way of oncolytic reactivation of KSHV [9].

Increasing data show dual inhibition of HDACs and BRD4 as a promising strategy against many tumors, such as pancreatic ductal adenocarcinoma [28] and gallbladder cancer [29], however, the impacts of dual HDACs/BRD4 inhibition on virus-associated lymphomas including PEL remain unclear. In the current study, we for the first time reported the synergistic effect of (+)-JQ1 with entinostat or SAHA on KSHV reactivation. Considering the potential advantages of single dual-targeting drug over the combination of two individual drug in the context of pharmacokinetic profile, drug-drug interactions and dosing scheduling [30], we further designed and synthesized a series of new dual HDACs/BRD4 inhibitors, which were generated by hybridizing the key binding moieties of (+)-JQ1 and entinostat or SAHA. After the screening, we identified a pair of epimers, 009P1 and 009P2, displaying effective growth inhibition of KSHV-associated lymphomas at low concentrations. Mechanistic studies of these compounds confirmed their dual inhibition of HDACs and BRD4 to induce virus lytic reactivation, cell cycle arrest and apoptosis from lymphoma cells. Furthermore, our preclinical data demonstrated that both compounds were orally bioavailable and dramatically suppressed tumor progression without visible toxicity in xenograft animal models, superior to JQ-1 or SAHA alone. Our data strongly supports a novel oncolytic strategy for improving the treatment of virus-associated lymphomas.

## Results

### The synergistic viral lytic-inducing effect of dual inhibition of HDACs/BRD4 and identification of newly synthesized dual inhibitors with anti-PEL activities

**Fig 1:**
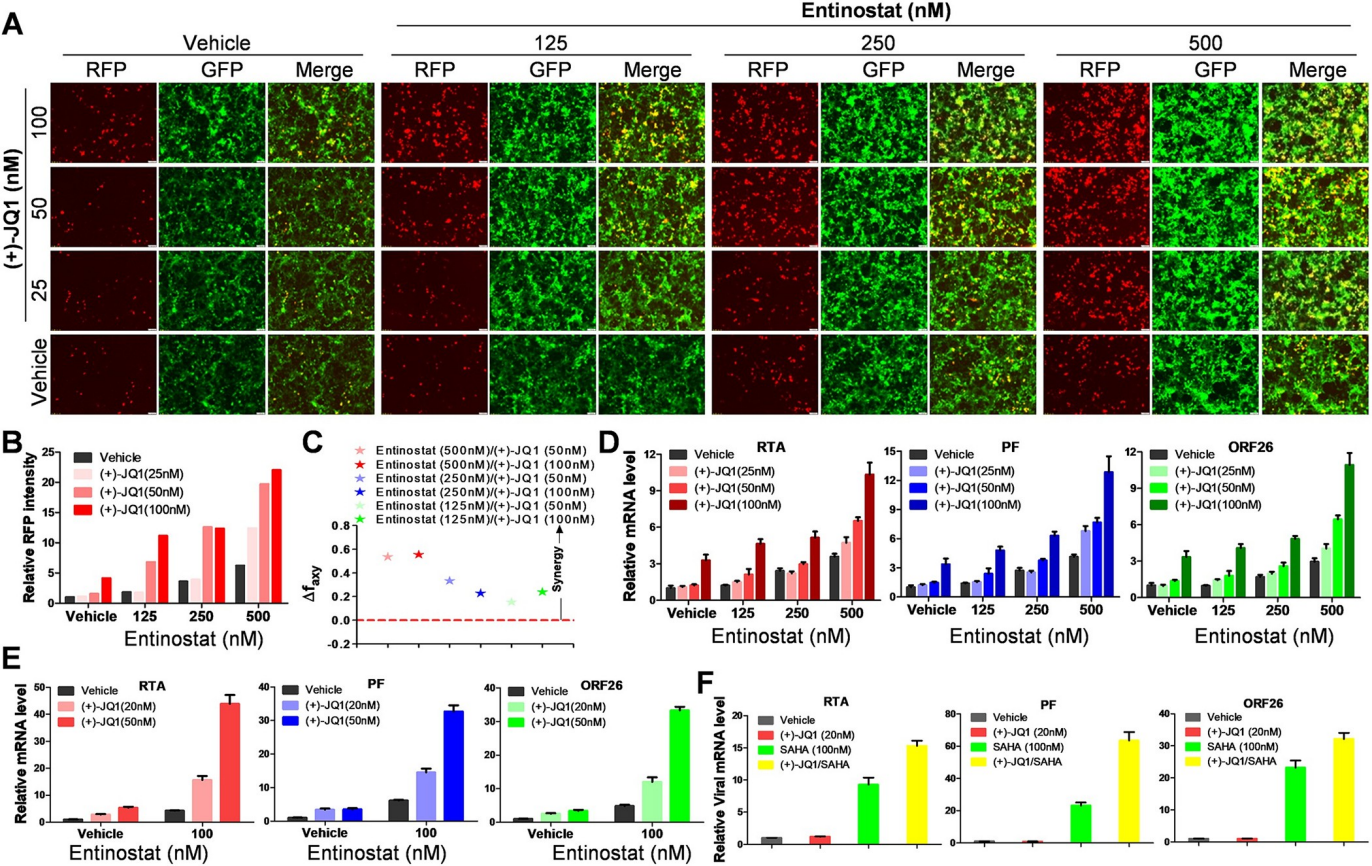
The synergistic viral lytic-inducing effect of dual inhibition of HDACs/BRD4. The iSLK.219 cells were exposed to compound combinations with low dose of Dox addition (0.05 μg/mL) for 48 h, then RFP expression was detected using fluorescence microscopy (**A**), and the fluorescence intensity was quantified using the software CellSens Ver.2.2. (**B**). The calculation of synergy for drug combination was analyzed by the Bliss independence model as described in the Methods (**C**). Synergy is defined as Δ*fa*_*xy*_>0 while Δ*fa*_*xy*_= or <0 indicates additive effect or antagonism. The expression of viral lytic genes was measured by RT-qPCR (**D**). Data was normalized as the fold change compared to the Dox control. BCBL-1 cells were exposed to a combination of entinostat /(+)-JQ1 (**E**) or SAHA/(+)-JQ1 (**F**) for 48 h, then the expression of lytic genes was measured by RT-qPCR. Data was normalized as the fold change compared to the DMSO control.

Previous studies reported lytic-inducing activities of HDACs or BRD4 inhibitors in KSHV latently infected cells [19,27]. Here, we sought to determine whether the combination of these inhibitors may have a synergistic effect on KSHV lytic reactivation. Initially, we used the human iSLK.219 cells to determine this hypothesis as described previously [31]. The cell line carries a recombinant rKSHV.219 virus encoding a constitutive GFP reporter and an RTA-inducible RFP reporter in the viral genome, thereby facilitating the monitoring of viral maintenance and lytic reactivation [32]. The iSLK.219 cells were treated with the HDACs inhibitor, entinostat, in combination with the BRD4 inhibitor, (+)-JQ1 in the presence of a low dose of doxycycline (Dox) for 48 h, and then RFP expression was imaged by the fluorescence microscopy. As shown in Fig 1A, we observed that when compared with Dox alone induction, treatments of either (+)-JQ1 or entinostat showed dose-dependently increased RFP expression. In contrast, their combination dramatically promoted the levels of RFP expression, much higher than their treatment alone and even the simple sum when used individually. The synergetic effects were then confirmed from the quantification of the fluorescence intensity (Fig 1B). The Δ*fa*_*xy*_ index analysis [33,34] showed that the values of almost all the dose combinations were more than 0, indicating a synergetic effect of such drug combinations (Fig 1C). Next, using RT-qPCR, we found that the combination treatments drastically elevated the expression of viral lytic genes from iSLK.219 cells, including RTA (Immediate-early gene), PF (Early gene) and ORF26 (Late gene), when compared to single treatments (Fig 1D). These findings indicate the synergistic effect of entinostat and (+)-JQ1 on KSHV reactivation. Next, we confirmed that such combinations also significantly elevated the expression of lytic genes from PEL cells (Fig 1E). Furthermore, the combination of (+)-JQ1 with another HDAC inhibitor, SAHA, also significantly increased the expression of lytic genes from BCBL-1 cells (Fig 1F). Taken together, all of these data strongly demonstrate the synergistic viral lytic-inducing effect of dual inhibition of HDACs and BRD4, which may represent a promising strategy for treatment of KSHV-associated malignancies.

**Fig 2:**
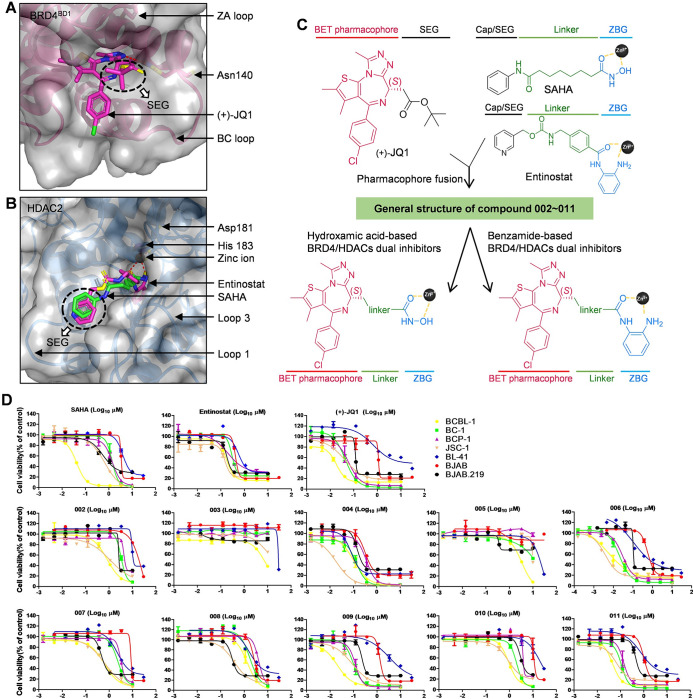
Identification of newly synthesized dual inhibitors with anti-PEL activities. (**A**) The binding mode of (+)-JQ1 with BRD4^BD1^ (PDB: 3MXF). BRD4^BD1^ is shown as hot pink cartoon with gray surface. Asn140 in BRD4^BD1^ is shown as sticks and colored in following pattern: atom C hot pink, atom N dark blue, and atom O yellow. (+)-JQ1 is shown as sticks and colored in following patten: atom C hot pink, atom N dark blue, atom O yellow, atom S golden, and atom Cl green. The solvent exposed region (SEG) of (+)-JQ1 are indicated with white arrow. (**B**) The binding modes of SAHA and entinostat with HDAC2 (PDB: 3MAX). HDAC2 is shown as blue cartoon with gray surface. Asp181 and His183 in HDAC2 are shown as sticks and colored in following pattern: atom C blue, atom N dark blue, and atom O yellow. The catalytic zinc ion in the active site of HDAC2 is shown as black sphere. SAHA is shown as sticks and colored in following pattern: atom C green, atom N dark blue, and atom O yellow. Entinostat is shown as sticks and colored in following pattern: atom C hot pink, atom N dark blue, and atom O yellow. The SEG of SAHA or entinostat is indicated with white arrow. Hydrogen bonds (H-bonds) are presented by red dash lines. (**C**) The HDACs/BRD4 dual inhibitors 002-011 were designed by fusing the key pharmacophores of (+)-JQ1 (pink) and HDAC inhibitors (blue), SAHA or entinostat, via various chemical linkers (green). (**D**) Anti-PEL activities of the new compounds. Five KSHV positive and two KSHV negative lymphoma cell lines were treated with indicated concentrations of these compounds for 72 h. Then the cytotoxicity was determined using the WST-1 assay. (+)-JQ1, SAHA and entinostat were used for the comparisons as single inhibitors. The CC_50_ for each compound was calculated using GraphPad Prism 5.0 software. Error bars represent S.D. for 3 independent experiments.

Considering the limitations of existing drugs and the advantages of a single dual-target drug over the conventional combination of two drugs, we designed and synthesized a series of new HDACs/BRD4 dual inhibitors 002-011, which were generated adducts of reported pharmacophores of (+)-JQ1 [35] (Fig 2A) and the entinostat analog [36] or SAHA [37] (Fig 2B) via an ester linker, due to the structural similarity with predecessor (+)-JQ-1 and its readiness to penetrate cells membranes [38] (Fig 2C). Then, five KSHV infected lymphoma cell lines (including BJAB.219 and 4 PEL cell lines, BCBL-1, BC-1, BCP-1, JSC-1) and two KSHV negative lymphoma cell lines (BJAB and BL-41) were used to screen their anti-PEL activities. As shown in Fig 2D, most of these compounds showed excellent inhibition of BJAB.219 or PEL cell growth in a dose-dependent manner (50% cytotoxicity concentrations, CC_50s_ at nM levels), which are either close to or even lower than CC_50_ of entinostat, SAHA or (+)-JQ1 (Table 1). Notably, most of these compounds showed much less effective on BJAB and BL-41 cells, indicating high selectivity on KSHV infected lymphoma cells and higher safety window.

**Table 1:** Screening of Anti-KSHV+ lymphoma activities of newly synthesized HDACs/BRD4 dual inhibitors.

Drugs	KSHV negative lymphomas (CC_50_ μM)^a^		KSHV-infected lymphomas (CC_50_ μM)				
	BL-41	BJAB	BJAB.219	BCBL-1	BC-1	BCP-1	JSC-1
SAHA	3.94	3.34	0.56	0.041	1.38	1.42	0.68
Entinostat	0.57	0.42	0.18	0.17	0.30	0.20	0.15
JQ1	0.68	1.11	~0.12	0.018	0.044	0.059	0.041
002	~ ^**b**^ 9.64	26.9	~2.57	0.82	3.28	~4.36	1.12
003	~15	>30	>10	7.46	>10	>10	>10
004	0.081	0.32	0.15	0.10	0.13	0.36	0.019
005	27.65	>30	>10	3.37	>10	>10	>10
006	0.11	0.57	0.10	0.0052	0.021	0.030	0.0039
007	2.30	8.52	0.47	0.62	2.35	3.16	0.66
008	~1.10	2.57	0.31	1.38	1.77	4.14	0.38
009	3.37	1.32	0.23	0.020	0.096	0.12	0.084
010	20.08	~11.04	2.94	0.93	2.04	~3.80	0.89
011	0.77	1.03	0.45	0.045	0.11	0.14	0.067

a. The CC_50_ represents the 50% cytotoxic concentration

b. The ‘~’ represents ‘approximately equal to’

### Compound 009 treatment strongly induces KSHV lytic replication, cell cycle arrest and apoptosis in PEL cells

**Fig 3:**
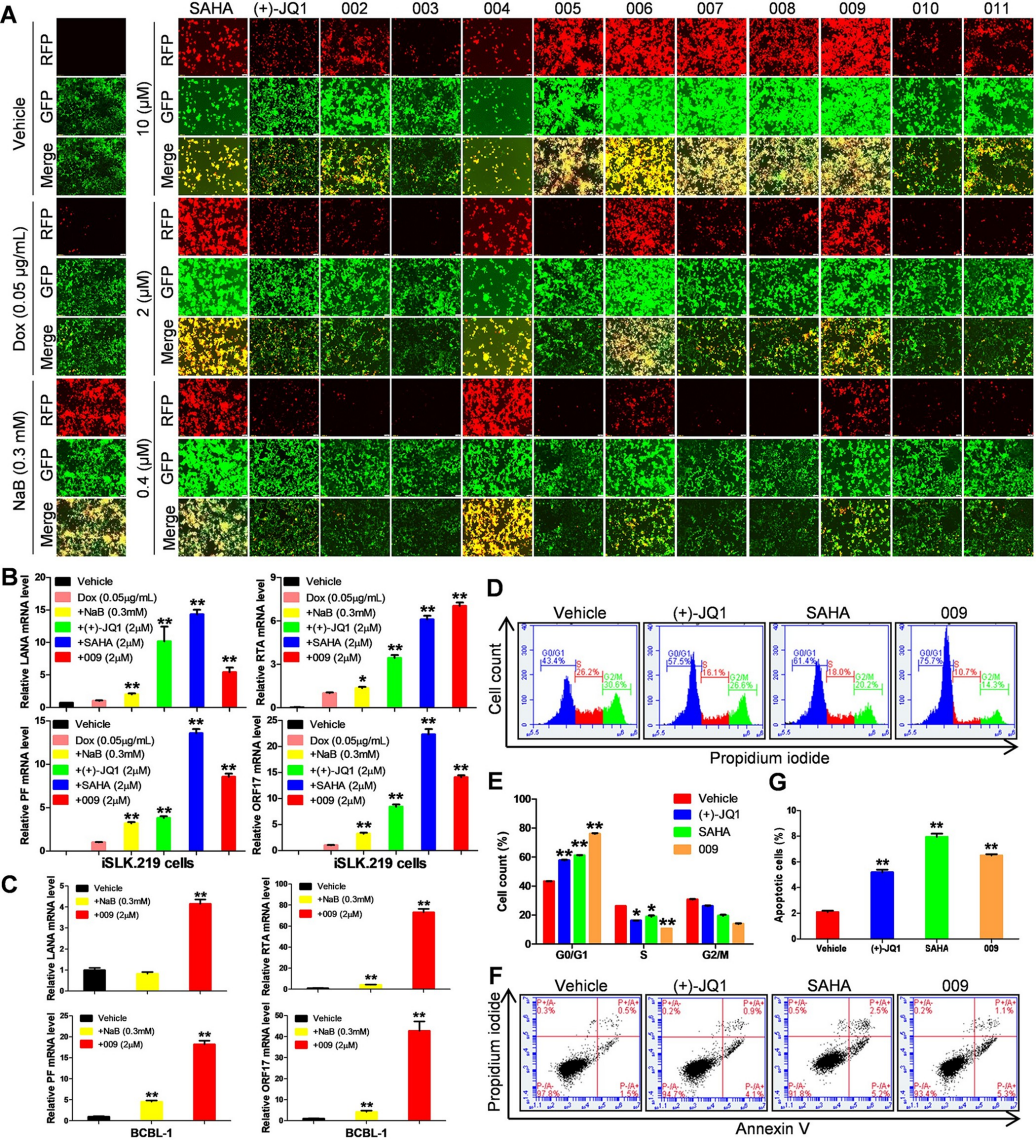
The new dual inhibitor 009 induces viral lytic reactivation, PEL cell cycle arrest and apoptosis. (**A**) The iSLK.219 cells were treated by indicated concentrations of compounds in conjunction with Dox (0.05 μg/mL), then RFP expression was evaluated at 72 h post-treatment as a measure of lytic reactivation. Compounds were incubated with the iSLK.219 cells in addition to Dox (0.05 μg/mL) (**B**) or BCBL-1 cells (**C**) for 72 h, then the transcripts of viral genes were measured by RT-qPCR. (**D-G**) BCBL-1 cells were exposed to the compounds at 2 × CC_50_ for 24 h, then cell cycle and apoptosis were measured by using flow cytometry analysis. NaB, SAHA and (+)-JQ1 were used as positive control. Error bars represent S.D. for 3 independent experiments, * = p<0.05; ** = p<0.01 (vs the vehicle control or Dox alone).

Next, we treated iSLK.219 cells with these new inhibitors to examine their KSHV lytic-inducing abilities. As shown in Figs 3A and S1, similar to NaB, a classical chemical inducer, all of these new inhibitors strongly induced RFP expression when compared to that of Dox alone group. However, in comparison to NaB leading to a pronounced increase in mature virion production, these new dual inhibitors caused less virion production when compared to Dox alone group from viral infected HEK293T cells by using immunofluorescence and qPCR assays (S2 Fig). The non-uniformity for high-levels of viral reactivation with low-levels of mature virion production following treatment with these inhibitors might be due to a cellular viral emergency response that triggers cell death signals, such as apoptosis [39]. Among these new inhibitors, compound 009 was selected for subsequent experiments due to the high efficacy and selectivity (Table 1). Using RT-qPCR, similar to (+)-JQ1, SAHA and NaB, compound 009 treatment elevated the expression of lytic genes, RTA, PF and ORF26, when compared to those of Dox alone group from iSLK.219 cells (Fig 3B). Consistent with these results, treated BCBL-1 cells also displayed increased lytic gene expression (Fig 3C). Similar to (+)-JQ1 and SAHA, compound 009 treatment led to a prominent cell cycle arrest of BCBL-1 at G1 phase (Fig 3D and 3E), and cell apoptosis (Fig 3F and 3G), using the flow cytometry analysis. Taken together, our data indicate that the inhibitory abilities of compound 009 against PEL cells are associated with virus lytic induction, cell cycle arrest and apoptosis.

### Compound 009 stereoisomers 009P1 and 009P2 exhibit anti-PEL activities via dual inhibition of HDACs/BRD4 signaling

**Fig 4:**
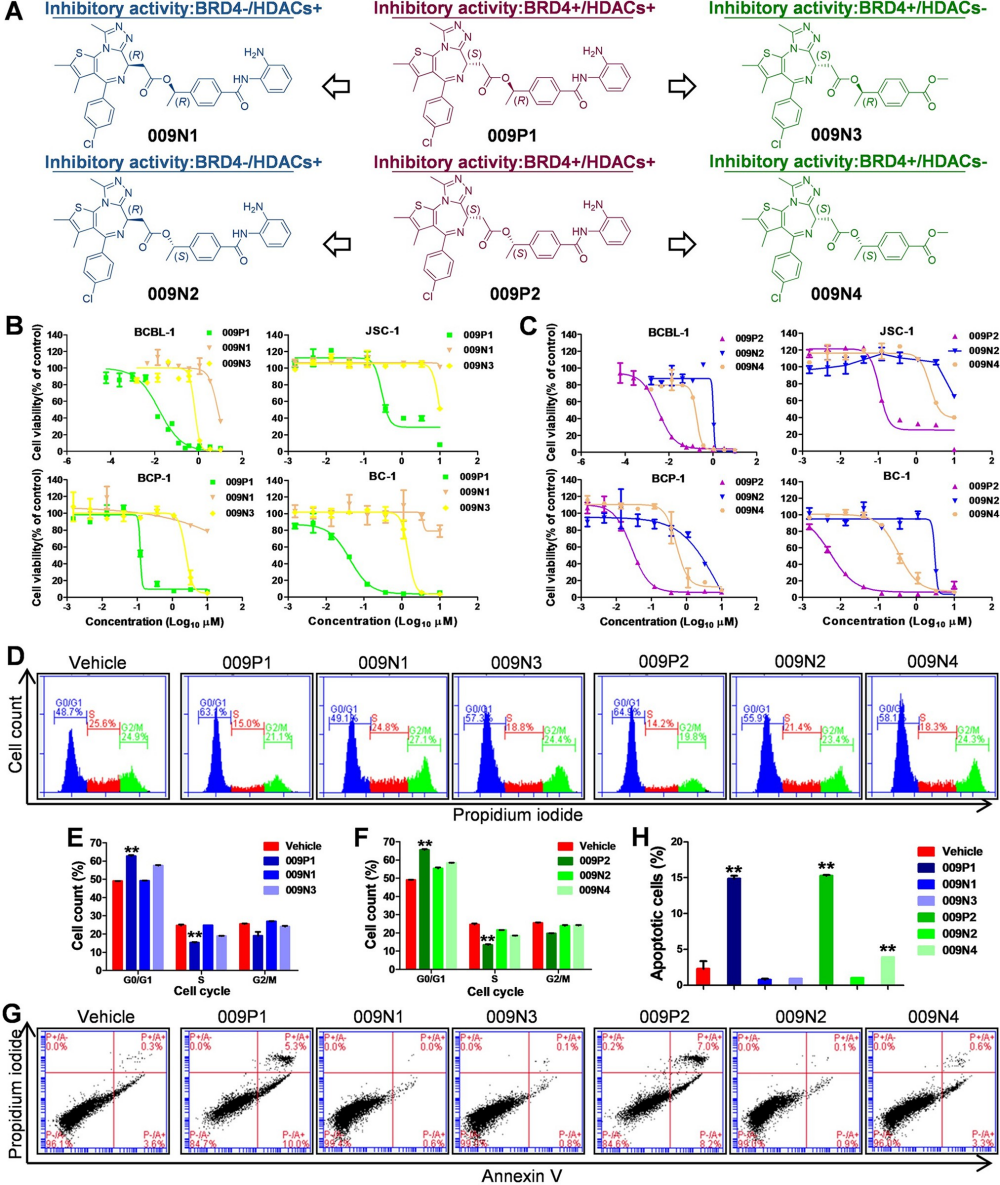
Identification of anti-PEL activities for compound 009 stereoisomers. (**A**) The chemical structures of anti-HDACs/BRD4 bifunctional molecules 009P1 and 009P2, as well as anti-HDACs or anti-BRD4 mono-functional molecules 009N1, 009N2, 009N3, and 009N4. (**B-C**) Four KSHV+ PEL cell lines were treated with indicated concentrations of 009P1, 009P2 or 009N1-N4 for 72 h, then the cytotoxicity was determined using the WST-1 assay. (**D-I**) BCBL-1 cells were treated with these compounds at the respective 2× CC_50_ for 24 h, then cell cycle (D-F) and apoptosis (G-I) were measured by flow cytometry analysis. Error bars represent S.D. for 3 independent experiments, ** = p<0.01 (vs vehicle control).

Compound 009 contains the pharmacophoric moiety of (+)-JQ1 bearing a chiral center with the *S* configuration synthesized as described in S3A Fig. Note here that 009 was also inserted with an additional chiral center at the phenylethyl acetyl ester linker with the *R* configuration for 009P1 and the *S* configuration for 009P2 (Fig 4A), respectively. 009P1 and 009P2 were synthesized as described in S3B and S3C Fig. The computational modeling docked both 009P1 and 009P2 nicely into the binding cavity of presentative BRD4^BD1^ or HDAC2 in a similar manner with *N*-(2-aminophenyl)benzamides for HDAC2 [36] or (+)-JQ1 for BRD4^BD1^ [35,37], respectively (S4 Fig), indicating that either 009P1 or 009P2 could bear the dual inhibitory activity on HDACs and BRD4. To further determine the anti-BRD4 and anti-HDACs bifunctional roles of 009P1, we also deigned and synthesized 009N1 with the inactive (-)-JQ1 pharmacophoric moiety with the *R* configuration to completely remove the activity on BRD4 [35], but retain the remaining structural component in 009P1 for the identical HADC activity (S3D Fig). While for 009N3, we removed the zinc binding benzenediamine in 009P1 to eliminate the activity on HDAC [40], but retains the (+)-JQ1 moiety for the identical activity on BRD4 (S3E Fig). Similarly, based on 009P2, 009N2 and 009N4 was generated as a pair of HDACs+/BRD4- and HDACs-/BRD4 mono-functional molecules (Figs 4A, S3D and S3E) for comparison. Initially, we found that both 009P1 and 009P2 were highly cytotoxic in different PEL cells at nM levels with CC_50_s similar to or even lower than 009. In contrast, all the 4 designed mono-functional molecules showed much less active in all of tested KSHV+ PEL cell lines (Fig 4B and 4C and Table 2), confirming the importance of dual inhibition. Moreover, our flow cytometry data showed that treatment with either 009P1 or 009P2 caused a higher degree of PEL cell cycle arrest (Fig 4D–4F) and cell apoptosis (Fig 4G–4I) than 009N1-009N4 treated cells. In addition, both 009P1 and 009P2 had much less cytotoxicity in primary cells such as HUVEC, a human primary endothelial cell line, while (+)-JQ1, entinostat and SAHA caused moderate cytotoxicity on these normal cells (S5 Fig). These data indicate the potentially safer and more effective anti-PEL activities of our new dual inhibitors *in vitro* compared to (+)-JQ1, entinostat or SAHA.

**Table 2:** Cytotoxicity of 009 stereoisomers against PEL cells.

Drugs	CC_50_ (μM)			
	BCBL-1	BC-1	BCP-1	JSC-1
009P1	0.016	0.042	0.12	0.30
009N1	7.56	>10	>10	>10
009N3	0.67	1.47	2.43	9.84
009P2	0.0033	0.0055	0.028	0.11
009N2	1.07	3.12	7.68	>10
009N4	0.18	0.34	0.53	2.32

### Dual inhibitors 009P1 and 009P2 induce lytic reactivation of KSHV

**Fig 5:**
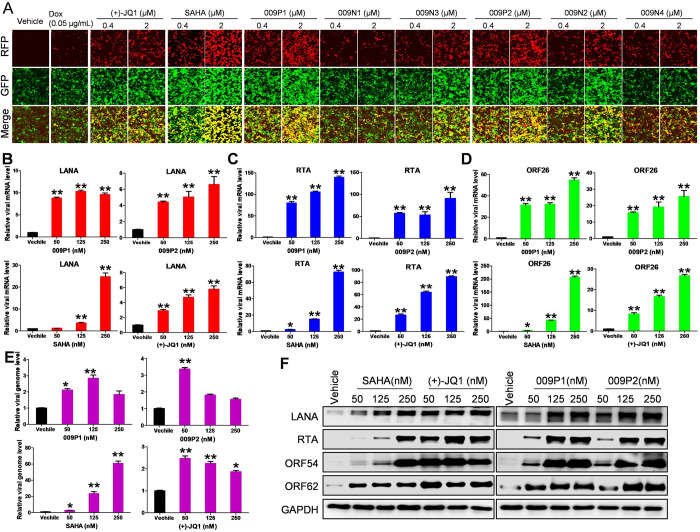
New dual inhibitors 009P1 and 009P2 treatments strongly induce KSHV lytic reactivation. (**A**) The iSLK.219 cells were treated by indicated concentrations of compounds in the addition to Dox (0.05 μg/mL) for 24 h, then the fluorescence intensity was detected via microscopy. (**B-F**) The transcripts of viral genes, viral DNA levels and protein expression were measured by using RT-qPCR, qPCR, and Western blot, respectively. SAHA and (+)-JQ1 were used as positive control. Error bars represent S.D. for 3 independent experiments, ** = p<0.01 (vs vehicle control).

To further explore the impacts of 009P1 or 009P2 on viral reactivation, the iSLK.219 cells were treated as described in the Methods. As shown in Figs 5A and S6, although all the compounds extensively promote RFP expression when compared to Dox alone group, treatment with these dual inhibitors produced much stronger RFP signals than those from any mono-functional molecule at the same doses. We then confirmed that both 009P1 and 009P2 treatment significantly increased expression of viral genes, such as latent gene, LANA and two lytic genes, RTA and ORF26, in a dose-dependent manner from PEL cells using RT-qPCR (Fig 5B–5D). Interestingly, both 009P1 and 009P2 treatment slightly increased viral DNA levels regardless of doses, which was more like (+)-JQ1, while SAHA obviously increased viral DNA (Fig 5E). These data demonstrate an alternative lytic replication with potent induction of viral gene expression and modest viral DNA synthesis in PEL cells treated with either 009P1 or 009P2. Using WB assays, we confirmed that these compounds treatment strongly elevated the expression of LANA, RTA, ORF54 (an early gene), ORF62 (a late gene) from PEL cells even at low concentrations (Fig 5F).

### The impacts of 009P1 and 009P2 on HDACs/BRD4 signaling and transcriptomic profile in PEL cells

**Fig 6:**
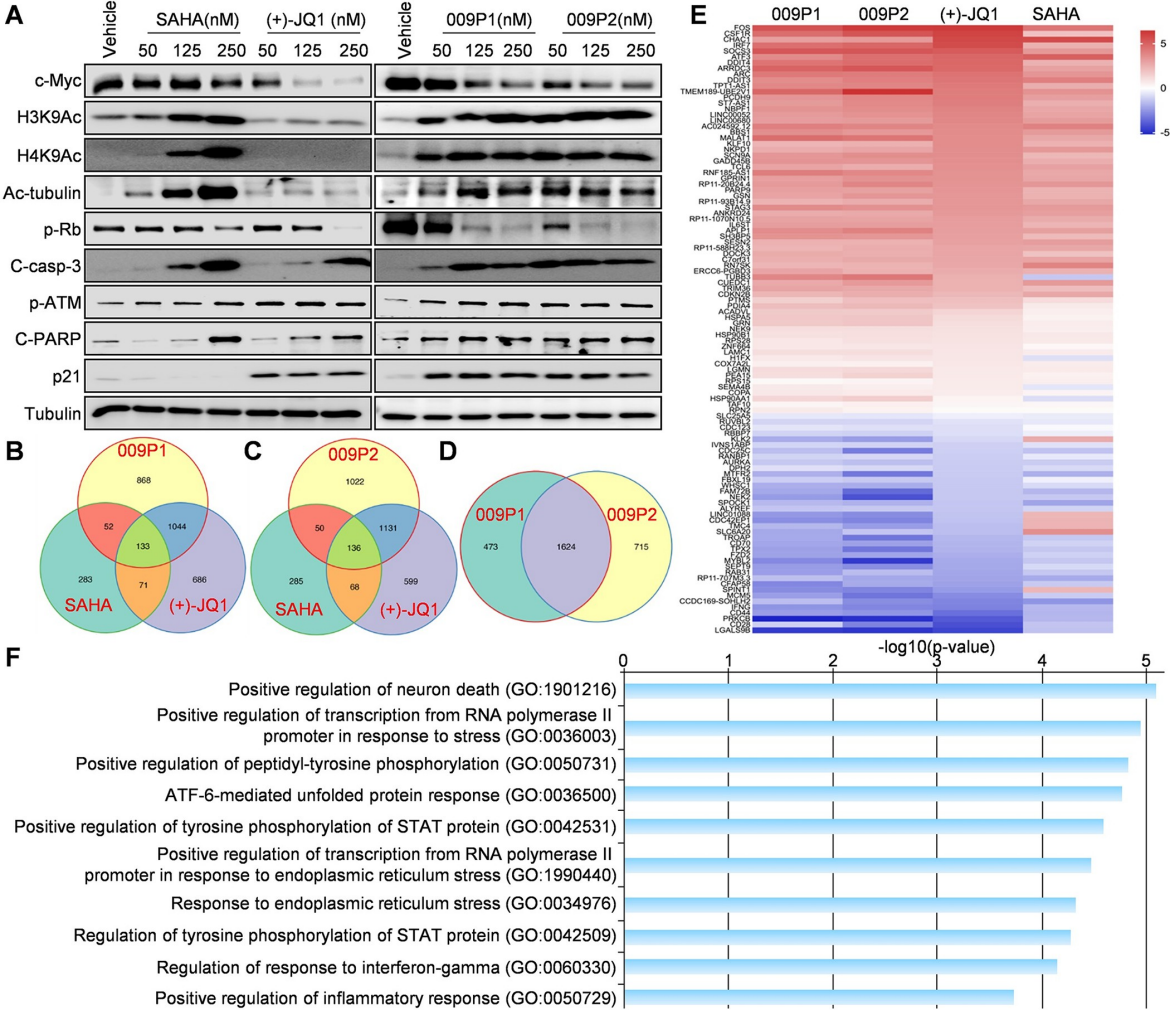
Molecular mechanisms for anti-PEL activities of dual inhibitors 009P1 and 009P2. (**A**) BCBL-1 cells were treated with indicated concentrations of compounds for 48 h, then protein expression was measured by Western blot. (**B-D**) The intersection analysis of significantly altered genes (expression change ≥ 2-fold and p<0.05) identified by RNA-Sequencing from 009P1-, 009P2-, (+)-JQ1-, or SAHA-treated BCBL-1 cells, respectively. (**E-F**) Heat map and enrichment analysis of genes commonly altered in 009P1-, 009P2-, (+)-JQ1-, and SAHA-treated BCBL-1 cells.

Previous studies have reported acetylation of histones and tubulin as the direct effectors in response to HDACs inhibition [41], and the expression of c-Myc is tightly regulated by BRD4 [42]. Our results showed that both 009P1 and 009P2 treatment simultaneously increased H3K9Ac, H4K9Ac and Acetyl-tubulin protein levels and decreased c-Myc expression from BCBL-1 cells (Fig 6A). In contrast, treatments with HDACs inhibitor SAHA or HDACs+/BRD4- mono-functional molecules 009N1 or 009N2 only upregulated the expression of H3K9Ac, H4K9Ac, Acetyl-tubulin but with subtle effect on c-Myc levels. The BRD4 inhibitor (+)-JQ1 or HDACs-/BRD4+ mono-functional molecules 009N3 and 009N4 drastically downregulated the expression of c-Myc (Figs 6A and S7). Collectively, our results demonstrate excellent and balanced activities blocking BRD4 and HDACs signaling by these new compounds.

Next, we evaluated whether 009P1 and 009P2 treatment altered the expression of several cell death regulatory proteins. We observed that both compounds decreased p-Rb and increased cleaved-caspase 3, cleaved-PARP, p-ATM and p21 in BCBL-1 cells (Fig 6A). Of note, the extent of these effector protein expressional changes were more pronounced in 009P1 and 009P2 treated cells relative to cells treated with (+)-JQ1 or SAHA. For example, at the concentration of 50 nM, both 009P1 and 009P2 strongly affected the expression of p-RB, cleaved-caspase 3 and cleaved-PARP, while which were almost unchanged under (+)-JQ1 or SAHA treatment. Also, the levels of p-RB under these dual inhibitors were extremely lower than those of mono-functional molecules at the same doses (S7 Fig), supporting the synergetic advantage of HDACs/BRD4 dual inhibition.

To further determine the global cellular changes induced by these new dual inhibitors, we compared the gene profile altered between vehicle- and compounds-treated BCBL-1 cells by using RNA-Sequencing analyses. Not surprisingly, an intersection analysis of the genes significantly upregulated or downregulated (≥ 2 fold-change and FDR < 0.05) showed that 009P1 and 009P2 displayed an overlapping gene expression pattern of altered genes when compared with (+)-JQ1 and SAHA, although there were more shared changes with (+)-JQ1 (Fig 6B and 6C). We found more than 2/3 gene changes were common in 009P1- and 009P2-treated PEL cells, but there were still 473 and 715 genes differentially expressed between 009P1- and 009P2-treated PEL cells, respectively (Fig 6D), indicating there are differences in down-stream effects. In addition, the heat map of all the most commonly altered genes altered in 009P1-, 009P2-, (+)-JQ1- and SAHA-treated BCBL-1 cells provided a clear visualization to support the downstream effects of 009P1 and 009P2 are more comparable to (+)-JQ1 (Fig 6E). The top 10 common genes upregulated or downregulated were listed in Table 3. GO enrichment analysis of these candidate genes indicated that several major functional categories were involved, such as the regulation of neuron death, response to endoplasmic reticulum stress and regulation of inflammatory response (Fig 6F).

**Table 3:** The top 10 common genes upregulated or downregulated from 009P1-, 009P2-, (+)-JQ1- and SAHA-treated BCBL-1 cells.

Gene Symbol	Description	Ratio			
		009P1	009P2	(+)-JQ1	SAHA
FOS	Fos proto-oncogene	46.1843361	77.0032785	78.6446424	21.0435465
CSF1R	Colony stimulating factor 1 receptor	24.8199607	42.3549988	62.4144717	3.86375708
CHAC1	ChaC glutathione specific gamma-glutamylcyclotransferase 1	8.34474292	9.24417266	55.6459477	53.9648398
IRF7	Interferon regulatory factor 7	23.7872529	28.8660963	52.6690518	5.62429866
SOCS3	Suppressor of cytokine signaling 3	41.4167037	41.8541836	37.1904631	9.78081638
ATF3	Activating transcription factor 3	12.8562272	22.9824435	28.7447949	33.6417674
DDIT4	DNA damage inducible transcript 4	21.7552086	24.9724967	28.3655982	5.42585182
ARRDC3	Arrestin domain containing 3	30.7196696	41.3188641	27.5845807	7.83410826
ARC	Activity regulated cytoskeleton associated protein	18.8145097	17.9087696	21.8273244	10.2613855
DDIT3	DNA damage inducible transcript 3	12.3317048	16.5256199	20.7303977	16.6183175
RP11-707M3.3	Novel transcript, antisense to MTFR1	0.34429926	0.23322861	0.22116076	0.24609911
CFAP58	Cilia and flagella associated protein 58	0.13580815	0.24736267	0.20892924	0.41099786
SPINT1	Serine peptidase inhibitor, Kunitz type 1	0.15036752	0.13111075	0.18756267	4.89304267
MCM5	Minichromosome maintenance complex component 5	0.20016521	0.09999881	0.14706319	0.42548904
CCDC169-SOHLH2	CCDC169-SOHLH2 readthrough	0.33581663	0.15107369	0.1299341	0.16965131
IFNG	Interferon gamma	0.16782267	0.13368986	0.1251487	0.31195761
CD44	CD44	0.19540363	0.16947509	0.09101497	0.29298696
PRKCB	Protein kinase C beta	0.02625854	0.03194942	0.06358254	0.30129685
CD28	CD28	0.4508938	0.12635371	0.05544176	0.37930477
LGALS9B	Galectin 9B	0.03811839	0.03508444	0.04586997	0.36050644

### Dual inhibitors 009P1 and 009P2 treatments effectively suppress PEL progression *in vivo*

**Fig 7:**
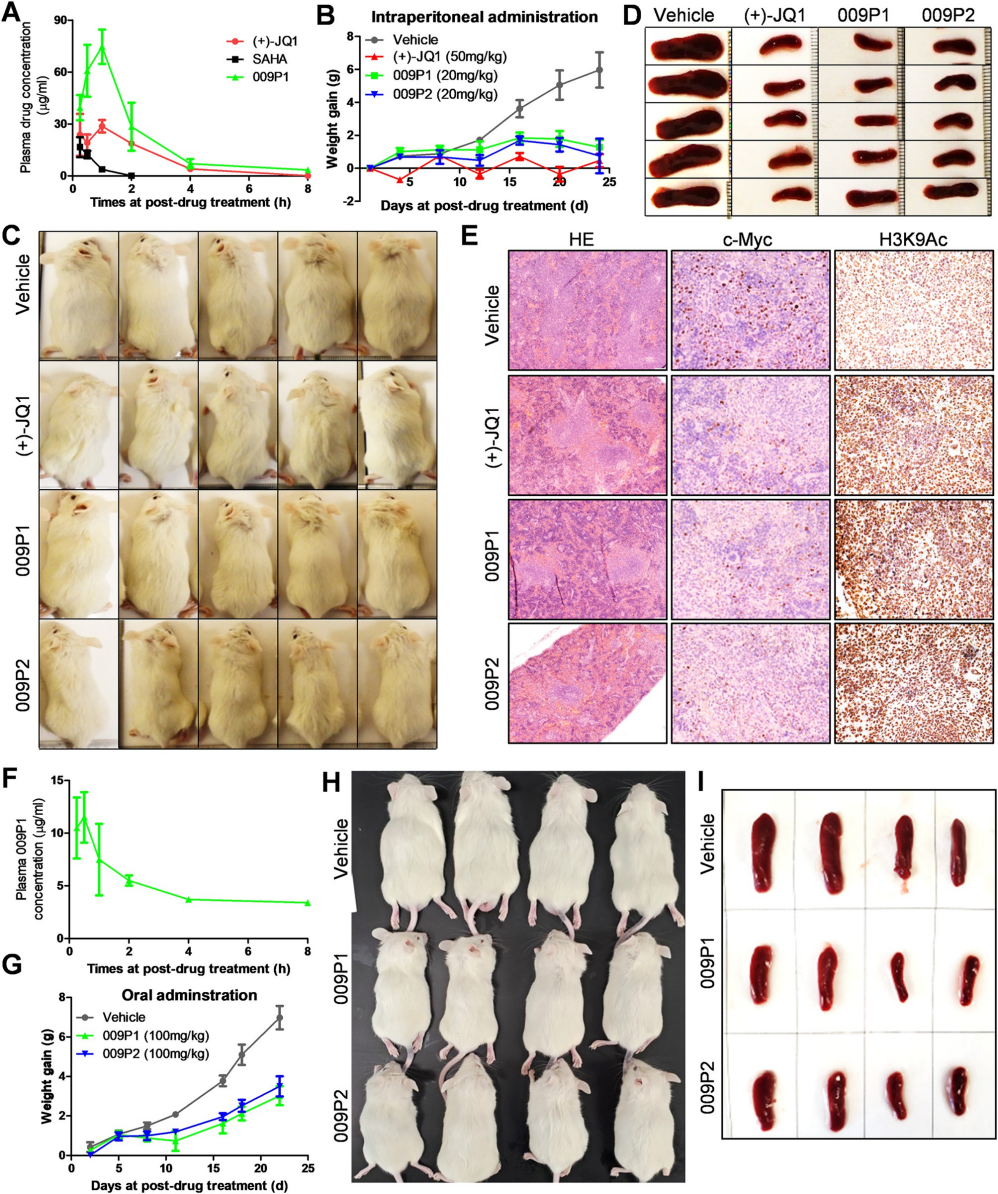
Treatments with 009P1 and 009P2 suppress PEL progression *in vivo*. (**A**) Plasma concentrations of (+)-JQ1, SAHA, or 009P1 after a single administration of 50 mg per kg body (i.p. injection) were presented as mean ± SD (n = 3 mice per time point). (**B-E**) NOD/SCID mice were injected i.p. with BCBL-1 cells. 72 h later, the compounds or vehicle were administered i.p. as described in the Methods, and weights were recorded weekly. At the end of the treatment period, the splenic tissues were collected from the vehicle or compound treated mice, compared and processed with the H&E and IHC staining. (**F**) Plasma concentrations of 009P1 after a single administration of 50 mg per kg body (oral gavage) were presented as mean ± SD (n = 3 mice per time point). (**G-I**) NOD/SCID mice were injected i.p. with BCBL-1 cells. 72 h later, the compounds or vehicle were administered orally as described in the Methods, and weights were recorded weekly.

First, pharmacokinetic (PK) properties of these new compounds were investigated in parallel by intraperitoneal (i.p.) dose at the concentration of 50 mg/kg. 009P1 exhibited adequate PK parameters with AUC_last_ of 159 h*μg/mL and terminal t_1/2_ of 1.66 h when compared to (+)-JQ1 (AUC_last_ of 78 h*μg/mL, terminal t_1/2_ of 2.27 h) (Fig 7A and Table 4). In contrast, SAHA was cleared too fast (AUC_last_ of 12 h*μg/mL, terminal t_1/2_ of 0.72 h) and therefore it was not used in the following antitumor efficacy studies. Then, the effects of (+)-JQ1 and both dual inhibitors 009P1 and 009P2 by i.p. administration on PEL tumor growth *in vivo* were tested by using an established murine xenograft model wherein PEL cells are introduced into the peritoneal cavity of immunocompromised mice [43,44]. As shown in Fig 7B–7D, 20 mg/kg of 009P1 and 009P2 both dramatically suppressed PEL tumor progression including reducing ascites formation and spleen enlargement over this timeframe, comparable to similar effects with 50 mg/kg of (+)-JQ1. Of note, 50 mg/kg of (+)-JQ1 showed visible toxicity in mice (abnormal weight loss in the curve shown in Fig 7B), which were not observed in either 009P1- or 009P2-treated mice. These data indicate a promising clinical utility of these new compounds. By using hematoxylin and eosin (H&E) staining, we observed substantial tumor infiltration into the spleen of vehicle-treated mice, whereas only diffuse small tumor nodules were apparent in the spleen of either 009P1- or 009P2-treated mice (Fig 7E). In contrast, the tumor infiltration was slightly reduced in the spleen of (+)-JQ1-treated mice. We detected a dramatic reduction in c-Myc expression, but an increased expression of H3K9Ac within spleen tissues from either 009P1- or 009P2-treated mice, when compared with those from vehicle-treated mice with IHC staining (Fig 7E).

**Table 4:** Pharmacokinetic studies of (+)-JQ1, SAHA, and 009P1 in mice.

PK parameters	Unit	(+)-JQ1 (i.p.)	SAHA (i.p.)	009P1 (i.p.)	009P1 (p.o.)
AUC_last_ ^a^	h*μg/mL	78	12	159	39
C_max_ ^b^	μg/mL	28.7	16.6	74.6	11.5
t_1/2_ ^c^	h	2.27	0.72	1.66	1.12
T_max_ ^d^	h	1	0.25	1	0.5
F^e^	%	NA^f^	NA	NA	24.5

a. AUC_last_ represents the area under the curve.

b. C_max_ represents the peak plasma concentration of drug in tested time points.

c. t_1/2_ represents the half-time of drug.

d. T_max_ represents the time point to C_max_.

e. Bioavailability F (%) represents the percentage value of AUC_last_ for p.o. / AUC_last_ for i.p.

f. NA means not available.

To explore the possibility of these dual inhibitors for oral (p.o.) administration, we evaluated the bioavailability (F%) and PK parameters of representative 009P1 in mice with single p.o. dose at 50 mg/kg. 009P1 showed efficient oral PK parameters with AUC_last_ of 39 h*μg/mL, half-life t_1/2_ of 1.12 h, and bioavailability (F) of 24.5% (Fig 7F and Table 4). Next, we found that oral administration of 009P1 and 009P2 still effectively repressed tumor burden in mice, and reduced ascites formation and spleen enlargement (Fig 7G–7I), indicating the potential for a more convenient way for drug administration in patients.

## Discussion

The oncolytic therapy through stimulating lytic reactivation of oncogenic herpesviruses to enhance selective killing of virus infected tumor cells has considerable potential as a novel strategy for treatment of virus-associated lymphomas [8–10]. In the current study, we for the first time identified the synergistic effect of HDACs and BRD4 inhibitors on KSHV lytic reactivation, although previous studies reported anti-PEL activities of these inhibitors alone through inducing viral reactivation [11,25]. In order to achieve effective and safety candidates based on a “two gene, one drug, one disease” paradigm, we designed and synthesized a series of novel HDACs/BRD4 dual inhibitors by fusing the key pharmacophores of (+)-JQ1 and entinostat analog or SAHA with an amide or ester bond as linker (Fig 2A–2C). Our initial screening showed most of these dual inhibitors selectively killed KSHV+ lymphoma cells at nanomolar levels (Fig 2D). One of these inhibitors, compound 009 with balanced inhibitory activity on HDACs and BRD4 from independent binding to each enzymes exhibited the synergistic and best anti-PEL potency, while much less toxic to KSHV negative lymphomas as expected (Table 1). Based on the computational modeling, the fused BRD4 and HDAC inhibitor 009 with relatively a smaller molecular size doesn’t allow to simultaneously bind to HDACs and BRD4, as the shared linker region is required for tight binding to both HDACs and BRD4. Furthermore, a pair of 009 epimers, 009P1 and 009P2, consistently displayed effective anti-PEL activities, and were less toxic to primary cells than conventional HDACs or BRD4 inhibitors, entinostat, SAHA and (+)-JQ1 (Table 2 and S5 Fig).

One of major mechanisms for high selectivity and efficacy of our dual inhibitors on KSHV+ lymphoma cells is likely due to enhance induction of viral lytic reactivation. By using two kinds of KSHV+ cells, PEL and iSLK.219, we confirmed that our dual inhibitors, particularly 009P1 and 009P2, strongly induced viral lytic gene expression (Figs 5 and S6). Interestingly, we observed lower mature virion production under the treatment of our dual inhibitors when compared to SAHA and (+)-JQ1 (Figs 3A and S2), suggesting these compounds induce an alternative lytic reactivation with an incomplete lytic phase. In fact, eliciting an incomplete lytic phase points towards a strong likelihood for a clinical benefit in that use of our new dual inhibitors in KSHV-infected patients may not cause viremia, which is a major concern for the oncolytic strategy [39,45]. One possible reason for this lack of achieving a full lytic cycle is that the rapid host apoptotic cell death induced by our compounds does not allow the virus to complete the lytic phase. In addition, we have observed that 009P1 and 009P2 treatments do not increase viral DNA replication even with high expression of lytic genes in PEL cells (Fig 5E). Although we do not know the underlying mechanisms yet, one interesting study reported that the overexpression of HDAC 1 and 6 was able to induce KSHV reactivation in PEL cells [20]. Another study reported an alternative apoptosis-triggered pathway, which was able to trigger KSHV reactivation, but only produce virion with lower infectivity [39].

Our RNA-Seq analysis has identified some interesting candidate genes potentially involved in the anti-PEL activities of 009P1 and 009P2 (Table 3). For example, DDIT3 and DDIT4, two DNA damage response (DDR) genes are highly upregulated in 009P1- and 009P2-treated PEL cells. Recent reports provide evidence that KSHV can activate the DDR during *de novo* infection of primary endothelial cells and this activation plays a role in establishing latency [46]. Another study demonstrated lytic reactivation of KSHV leads to activation of the ataxia telangiectasia mutated (ATM) and DNA-dependent protein kinase (DNA-PK) DDR kinases [47]. Interestingly, inhibition of ATM results in the reduction of overall levels of viral replication whereas inhibition of DNA-PK increases activation of ATM and leads to earlier viral release. Therefore, we are interested to explore the role of DDR and related proteins in anti-PEL activities of our new dual inhibitors in follow-up studies. In contrast, LGALS9B (Galectin 9B) represents one of the candidate genes, which are dramatically downregulated in 009P1- and 009P2-treated PEL cells. The functional role of LGALS9B remains unclear in KSHV pathogenesis, however, previous work found interactions between the galectin-1 (Gal-1) and specific target N-glycans link tumor hypoxia to neovascularization as part of the pathogenesis of KS [48]. Therapeutic administration of a Gal-1-specific neutralizing mAb attenuated abnormal angiogenesis and promoted tumor regression in mice bearing established KS tumors.

Our *in vivo* data showed our new dual inhibitors including 009P1 displayed acceptable PK parameters, whereas SAHA was rapidly cleared in mice (Table 4). The antitumor efficacy studies demonstrated that both oral and IP delivery of 009P1 and 009P2 at a relatively low dose (20 mg/kg) displayed dramatic suppression of PEL progression in mice without visible toxicity (Fig 7). Although high dose of (+)-JQ1 (50 mg/kg, IP) showed similar therapeutic efficacy, signs of toxicity appeared in these treated mice. Furthering these distinct toxicity profiles, we conducted a subsequent study via an oral administration of (+)-JQ1, 009P1, and 009P2 at 100 mg/kg, however, unfortunately (+)-JQ1 showed such severe toxicity in the mice we had to terminate the experiment based on our institute IACUC policies. Therefore, our *in vivo* data indicate a notable therapeutic advantage of these HDACs/BRD4 dual inhibitors over conventional mono-inhibitors.

Taken together, our study highlights the advantages of HDACs/BRD4 dual inhibitors as novel and promising agents against these virus-associated lymphomas, and strengthens the potential of an oncolytic strategy for improving treatments of these specific malignancies.

## Material and methods

### Ethics statement

All the animal protocols were approved by the University of Arkansas for Medical Sciences Animal Care and Use Committee (No. 3960) in accordance with national guidelines.

### Cell culture and reagents

BCBL-1 and iSLK.219 cells were kindly provided by Dr. Pinghui Feng (University of Southern California). BL-41, BJAB, BCP-1, BC-1, BC-3, JSC-1, HUVEC cells were purchased from American Type Culture Collection (ATCC), and cultured as recommended by the manufacturer. For making BJAB.219 cells, BJAB cells were infected with rKSHV.219 stock by spinoculation as previously reported by centrifugation at 1500×g for 60 min [31]. Then, puromycin (10 μg/ml) was added into the medium at 72 h post-infection and the medium containing puromycin was replaced twice a week. After 6-week high concentration of puromycin selection, BJAB.219 cells were then cultured in the medium with 0.5 μg/ml puromycin.

### Compounds synthesis and purifications

All compounds were synthesized and purified as described in S1 Supplementary Methods. All reactions were monitored by thin-layer chromatography (TLC) on silica gel plates. Flash chromatography was performed using silica gel (200−300 mesh). ^1^H-NMR spectral data were recorded on a Varian Mercury 400 NMR spectrometer, and ^13^C-NMR was recorded on a Varian Mercury 126 NMR spectrometer at ambient temperature. Chemicals shifts (*δ*) were reported in ppm, coupling constants (J) were in hertz, and the splitting patterns were described in follows: s for singlet; d for doublet; t for triplet; q for quartet; and m for multiplet. Mass spectrometry was conducted using a Thermo Fisher LCQ-DECA spectrometer (ESI-MS mode). All tested compounds were purified to ≥ 95% purity as determined by high performance liquid chromatography (HPLC). A Kinetex 5 μm XB-C18 100 Å LC column (150 * 4.6 mm) with an Agilent 1100A HPLC system was used for the determination of purity. The column was maintained at 37°C for the duration of HPLC. 009 and its analogs were synthesized and purified as described in S1 Supplementary Methods. Briefly, (+)-JQ-1/or (-)-JQ-1 acid was coupled with the pharmacophoric moiety of entinostat [36] or SAHA [37] by using benzotriazol-1-yloxytripyrrolidinophosphonium hexafluorophosphate (PyBOP) reagent [49] to get precursors of 009, 009P1, 009P2, 009N1 or 009N2. *N*,*N*-diisopropylethylamine was used as a base. Dimethylformamide was used as a solvent. The *tert*-butyloxycarbonyl (Boc) protection group was then removed using trifluoracetic acid in dichloromethane to generate 009, 009P1, 009P2, 009N1 or 009N2. For the synthesis of 009N3 and 009N4, (+)-JQ-1 acid was directly coupled with (*R*)- or (*S*)-methyl-4-(1-hydroxyethyl)benzoate respectively using the same coupling conditions as aforementioned.

### Molecular modeling procedure

The binding model for compounds 009P1 and 009P2 were generated based on two separated template crystal structures of BRD4^BD1^ (PDB code: 3MXF) [35] and HDAC2 (PDB code: 3MAX) [36] from the Protein Data Bank. Any sequence duplications were removed to provide a single entry. Schrodinger Glide’s Protein Preparation Wizard was used to prepare the raw structure with the following parameters: 1) maximize hydrogens at all amino acid residues, 2) remove any irrelevant water molecules, and 3) minimize energy of protein structures as whole. Subsequently, the existing ligands from template structures were utilized to create the receptor grids with all dimensions extended to 22Å. Chemical structures of 009P1 and 009P2 were imported and processed via LigPrep to maintain proper enantiomers. Both compounds were allowed to flexibly dock and evaluated for appropriate binding pose. Glide extra precision mode (Glide XP) was used for scoring. Additionally, various studies informed that chelation of zinc metal is critical for binding of benzamide functional group to HDAC [36], therefore, we utilized the metal coordination constrain while allowing free rotation of our candidate compounds. Figs 2A, 2B and S4 were generated with PyMOL version 2.5.

### Cytotoxicity assay

The cell viability following treatment with compounds was assessed by the WST-1 assay (Roche, Indianapolis, Indiana, USA) according to the manufacturer’s protocol as described previously [44]. The absorbance signal was measured using a microplate reader (Biotek Synergy 2). The 50% cytotoxic concentration (CC_50_) for each compound was calculated from these dose-response curves using the software GraphPad Prism v5.0. Data were normalized as the fold change compared to the DMSO control.

### Infectivity assays and fluorescence detection

The iSLK.219 cells latently carry a recombinant rKSHV.219 virus and a doxycycline (Dox)-inducible gene expression system for expression of viral replication and transcription activator (RTA) protein, of which expression is essential and sufficient for triggering KSHV reactivation [32]. The rKSHV.219 contains two fluorescent protein genes, the green fluorescent protein (GFP) and red fluorescent protein (RFP), which are derived from the EF-1α promoter and KSHV lytic PAN promoter, respectively [32]. iSLK.219 cells were employed to evaluate viral reactivation and infectivity as described previously [31]. The cells were treated by Dox (0.05 μg/ml) in combination with tested compounds at concentrations and time-points as indicated, then RFP expression was detected by a fluorescent microscopy (Olympus DP80) and quantitatively analyzed by the imaging software CellSens Ver.2.2. The rest of the supernatants were used to infect HEK293T by spinoculation as previously reported by centrifugation at 1500×g for 60 min [31], then GFP expression was detected at 48 h post-infection by fluorescent microscopy.

### Quantitative analysis of synergy of compound combinations

Bliss independence model was used to evaluate the viral lytic-reactivated activity of drug combinations as described previously with modification [33,34]. For drugs x and y, we used an equation Δ *fa*_*xy*_ *= fa*_*xy*_,_*O*_ *– fa*_*xy*,*P*_, where Δ *fa*_*xy*_ > 0, =0 or <0 provides an indication of synergy, additive effect or antagonism, respectively. The *fa*_*xy*_,_*O*_ is the observed fraction affected by the drug combination, and *fa*_*xy*,*P*_ represents the predicted fraction affected by the drug combination and is defined by the equation *fa*_*xy*,*P*_ *= fa*_*x*_ *+ fa*_*y*_ – (*fa*_*x*_ *× fa*_*y*_), where *fa*_*x*_*=* (*RFP intensity value from drug x / RFP intensity value from 0*.*05 μg/ml of Dox group*) */* (*the highest RFP intensity value from drug combinations / RFP intensity value from 0*.*05 μg/ml of Dox group*). The calculation of *fa*_*x*_ for iSLK.219 cells used relative RFP intensity, and the RFP intensity value of combination of entinostat (500 nM)/(+)-JQ1 (100 nM) was used as the denominator due to the highest value in these combination.

### Cell cycle and apoptosis analysis

Flow cytometry was used for the quantitative assessment of cell cycle and apoptosis [44]. Briefly, to measure cell cycle response, PEL cell pellets were fixed in 70% ethanol, and incubated at 4°C overnight. Cell pellets were re-suspended in 0.5 mL of 0.05 mg/mL PI plus 0.2 mg/mL RNaseA, and incubated at 37°C for 30 min. Cell cycle distribution was analyzed using a BD Accuri C6 flow cytometer. Apoptosis was assessed by the FITC-Annexin V/propidium iodide (PI) Apoptosis Detection Kit I (BD Pharmingen, San Jose, California, USA) on a flow cytometer. Data was normalized as the fold change compared to the DMSO control.

### Quantitative Reverse Transcription-PCR (RT-qPCR) and quantitative PCR (qPCR)

Transcripts of genes of interest were measured by RT-qPCR. Total cellular RNA was isolated and purified using the Qiagen RNeasy Kit (Qiagen, Germantown, Maryland, USA). The cDNA was synthesized using a High-Capacity cDNA Reverse Transcription Kit (Applied Biosystems). KSHV genomic DNA was isolated and purified using the QIAamp DNA mini Kit (Qiagen, Germantown, Maryland, USA) and was also measured by qPCR. All RT-qPCR and qPCR assays were performed using a Real-Time PCR detection system (C1000 touch thermal cycler, Bio-Rad) using the iTaq Universal SYBR Green Supermix (Bio-Rad) with specific primers (S1 Table) and analyzed as described previously [44].

### Western Blot (WB)

The expression of the proteins of interest was detected by Western Blot using protein-specific antibodies as described previously [44]. Immuno-reactive bands were identified using a Bio-rad Clarity Max Western ECL Substrate kit, and visualized by Bio-rad Chemi Doc Imaging System. Anti-LANA antibody was purchased from Advanced Biotechnologies Inc (Eldersburg, MD, USA). Antibodies for KSHV RTA and ORF54 were purchased from Helmholtz-Munich, Germany and anti-ORF62 antibody was purchased from Novus Biologicals (Centennial, CO, USA). Antibodies for c-Myc, H3K9Ac, H4K9Ac, cleaved Caspase3, cleaved PARP, p-Rb, p-ATM, acetyl-tubulin and p21 were obtained from Cell Signaling Technology (Danvers, MA, USA).

### Pharmacokinetic (PK) of compounds in mice

The PK profiles of compounds were determined in plasma following a single intraperitoneal (i.p.) injection (50 mg per kg body weight) or oral gavage (p.o.) administration (50 mg per kg body weight) to NOD/SCID mice, 6–8-week-old, male (Jackson Laboratory, Ellsworth, Maine, USA). The i.p. and p.o. dose were formulated with DMSO/PEG300/Tween80/saline (5/30/10/55). Samples were obtained at 0.25, 0.5, 1, 2, 4, and 8 hours post dosing via tail snipping, transferred into plastic microcentrifuge tubes containing 4 μL of K2-EDTA (0.5 M) as anti-coagulant and placed on wet ice until centrifugation. Harvested blood samples were centrifuged shortly after collection at 4,000 g 4°C for 10 minutes. After centrifugation, the amount of compounds in the plasma was determined using HPLC-coupled tandem mass spectrometry (LC-MS/MS). Values are calculated from arithmetic mean plasma concentrations (n = 3 mice per condition). AUC_last_ and t_1/2_ were determined using GraphPad Prism v5.0. The bioavailability F (%) = AUC_last_ for p.o. / AUC_last_ for i.p. × 100 [50].

### PEL xenograft model

In all NOD/SCID mice, 6–8-week-old, male (Jackson Laboratory, Ellsworth, Maine, USA), 1 × 10^7^ BCBL-1 cells in 200 μL RMPI-1640 without FBS were injected intraperitoneally and then mice were randomized into treatment groups of 6 mice as described previously [44]. The tested compounds or vehicle was administered initially at 72 h after BCBL-1 injections, and continued once daily, 2 days per week for 3 weeks. Weights were recorded weekly as a surrogate measure of tumor progression. At the end of experiment, the spleens of mice were excised for immunoblots and immunohistochemical analyses.

### RNA-Sequencing and enrichment analysis

RNA-Sequencing (RNA-Seq) of triplicate samples was performed by BGI Americas Corporation using their unique DNBSEQ sequencing technology. The completed RNA-Sequencing data was submitted to NCBI Sequence Read Archive (SRA# # PRJNA813422). Raw sequencing reads were analyzed using the RSEM software (version 1.3.0; human GRCh38 genome sequence and annotation) and gene expression was quantified as previously described [51]. The EBSeq software was utilized to call differentially expressed genes that were statistically significant using a false discovery rate (FDR) less than 0.05. Differentially expressed genes between compounds- and vehicle-treated PEL cells were used as input for the GO enrichment analyses.

### Immunohistochemistry

Formalin-fixed, paraffin-embedded tissues were microtome-sectioned to a thickness of 4 μm and placed on electromagnetically charged slides. Immunohistochemistry was performed as described previously [44], and the c-Myc and H3K9Ac antibodies were purchased from Abcam. Images were collected using an Olympus BX61 microscope equipped with a high resolution DP72 camera and CellSense image capture software.

## Supporting information

S1 Table Primer sequences for qPCR and RT-qPCR. (DOCX)

S1 Fig The effects of new dual inhibitors on KSHV reactivation. The iSLK.219 cells were treated with indicated concentrations of compounds with the addition of Dox (0.05 μg/mL) for 72 h, then the levels of RFP expressed cells were analyzed and quantified using flow cytometry. Error bars represent S.D. for 3 independent experiments, * = p<0.05, ** = p<0.01.
(TIF)

S2 Fig The effects of new dual inhibitors 002-011 on KSHV virion production. The iSLK.219 cells were treated by indicated concentrations of compounds with the addition of Dox (0.05 μg/mL) for 72 h treatment, then the supernatants were collected to infect HEK293T cells. (**A**) Cells were stained with DAPI at 48 h post-infection and the fluorescence signals were examined using fluorescence microscopy. (**B**) The viral DNA levels were quantified using qPCR as described in the Methods. Data was normalized as the fold change compared to the Dox control.
(TIF)

S3 Fig **Synthesis of 009 (A), 009P1 (B), 009P2 (C), 009N1/009N2 (D) and 009N3/009N4 (E).** Reagents and conditions: (a) (Boc)_2_O, NaOH, 1,4-dioxane, H_2_O, 0°C to rt, overnight; (b) 4-(1-hydroxyethyl)benzoic acid, HATU, DIPEA, DMF, 0°C to rt, overnight; (c) TFA, DCM, rt, overnight; (d) PyBOP, DIPEA, DMF, 0°C to rt, overnight; (e) TFA, DCM, 0°C to rt, 3 h; (f) LiOH·H_2_O, THF, MeOH, H_2_O, 0°C to rt, overnight; (g) **2**, HATU, DIPEA, DMF, 0°C to rt, overnight; (h) **5**, PyBOP, DIPEA, DMF, 0°C to rt, overnight; (i) TFA, DCM, 0°C to rt, 2-3 h; (j) **9**, PyBOP, DIPEA, DMF, 0°C to rt, overnight; (k) **13**, PyBOP, DIPEA, DMF, 0°C to rt, overnight; (l) **7**, PyBOP, DIPEA, DMF, 0°C to rt, overnight; (m) **11**, PyBOP, DIPEA, DMF, 0°C to rt, overnight.
(TIF)

S4 Fig Molecular modeling of 009P1 and 009P2 with BRD4^BD1^ or HDAC2. (**A**) The binding mode of 009P1 and (+)-JQ1 with BRD4^BD1^. BRD4^BD1^ is shown as hot pink cartoon with gray surface. Asn140 in BRD4^BD1^ is shown as sticks and colored in following pattern: atom C hot pink, atom N dark blue, and atom O yellow. (+)-JQ1 is shown as sticks and colored in following pattern: atom C hot pink, atom N dark blue, atom O yellow, atom S golden, and atom Cl green. 009P1 is shown as sticks and colored in following pattern: atom C light purple, atom N dark blue, atom O yellow, atom S golden, and atom Cl green. (**B)** The binding mode of 009P2 and (+)-JQ1 with BRD4^BD1^. BRD4^BD1^ is shown as hot pink cartoon with gray surface. Asn140 in BRD4^BD1^ is shown as sticks and colored in following pattern: atom C hot pink, atom N dark blue, and atom O yellow. (+)-JQ1 is shown as sticks and colored in following pattern: atom C hot pink, atom N dark blue, atom O yellow, atom S golden, and atom Cl green. 009P2 is shown as sticks and colored in following pattern: atom C dark green, atom N dark blue, atom O yellow, atom S golden, and atom Cl green. (**C**) The binding mode of 009P1 and SAHA with HDAC2. HDAC2 is shown as blue cartoon with gray surface. Asp181, His183, and Phe155 in HDAC2 are shown as sticks and colored in following pattern: atom C blue, atom N dark blue, and atom O yellow. 009P1 is shown as sticks and colored in following pattern: atom C light purple, atom N dark blue, atom O yellow, atom S golden, and atom Cl green. The catalytic zinc ion in the active site of HDAC2 is shown as black sphere. SAHA is shown as sticks and colored in following pattern: atom C green, atom N dark blue, and atom O yellow. (**D**) The binding mode of 009P2 and vorinostat with HDAC2. HDAC2 is shown as blue cartoon with gray surface. Asp181, His183, and Phe155 in HDAC2 are shown as sticks and colored in following pattern: atom C blue, atom N dark blue, and atom O yellow. 009P2 is shown as sticks and colored in following pattern: atom C dark green, atom N dark blue, atom O yellow, atom S golden, and atom Cl green. The catalytic zinc ion in the active site of HDAC2 is shown as black sphere. SAHA is shown as sticks and colored in following pattern: atom C green, atom N dark blue, and atom O yellow. Hydrogen bonds (H-bonds) are depicted as red dash lines.
(TIF)

S5 Fig Cytotoxicity of tested compounds on HUVEC. The primary cells HUVEC were treated with indicated concentrations of compounds for 72 h, then cell viability was assessed using the WST-1 assay. The CC_50_ for each compound was calculated from the dose-response curves using Graphpad Prism 5.0 software. Data was normalized as the fold change compared to the DMSO control.
(TIF)

S6 Fig The effects of new dual inhibitors 009P1 and 009P2 on KSHV reactivation. The iSLK.219 cells were treated with indicated concentrations of compounds in the addition to Dox (0.05 μg/mL) for 24 h, then, the levels of RFP expressed cells were examined (**A**) and quantified (**B**) using flow cytometer. Error bars represent S.D. for 3 independent experiments, ** = p<0.01.
(TIF)

S7 Fig The impacts of mono-functional molecules on targeted proteins expression in PEL cells. The BCBL-1 cells were treated with indicated concentrations of compounds for 48 h, then protein expression was determined using Western blot assays.
(TIF)

S1 Supplementary Methods Supplementary methods about chemicals synthesis. (DOCX)
