# Supplementary material for: Oncolytic strategy using new bifunctional HDACs/BRD4 inhibitors against virus-associated lymphomas
Source: PLoS Pathog. 2023 Jan 13;19(1):e1011089. doi: 10.1371/journal.ppat.1011089 (PMC9879403; doi:10.1371/journal.ppat.1011089)
Supplement: S1 Table — (DOCX) [file ppat.1011089.s001.docx]

|  | **Gene** | **Forward primers** | **Reverse primers** |
| --- | --- | --- | --- |
| qPCR | LANA | 5'-TCCCTCTACACTAAACCCAATA-3' | 5'-TTGCTAATCTCGTTGTCCC-3' |
|  | GAPDH | 5'-GCTCCCTCTTTCTTTGCAGCAAT-3' | 5'-TACCATGAGTCCTTCCACGATAC-3' |
| RT-qPCR | LANA | 5'-TCCCTCTACACTAAACCCAATA-3' | 5'-TTGCTAATCTCGTTGTCCC-3' |
|  | RTA | 5’-CACAAAAATGGCGCAAGATGA-3’ | 5’-TGGTAGAGTTGGGCCTTCAGTT-3’ |
|  | PF | 5’-CGAGTCTTCGCAAAAGGTTC-3’ | 5’-AAGGGACCAACTGGTGTGAG-3’ |
|  | ORF26 | 5’-GCTCGAATCCAACGGATTTG -3’ | 5’- AATAGCGTGCCCCAGTTGC-3’ |
|  | ORF17 | 5’-AGATTTTTCACGGGGGCTCTGG-3’ | 5’- TGGGCTGGACACTGGGTCTATTTC-3’ |
|  | β-actin | 5’-ATCGTGCGTGACATTAAGGAG-3’ | 5’-GGAAGGAAGGCTGGAAGAGT-3’ |

**S1 Table. Primer sequences for qPCR and RT-qPCR**
