# Supplementary figures and images for: Oncolytic strategy using new bifunctional HDACs/BRD4 inhibitors against virus-associated lymphomas
Source: PLoS Pathog. 2023 Jan 13;19(1):e1011089. doi: 10.1371/journal.ppat.1011089 (PMC9879403; doi:10.1371/journal.ppat.1011089)

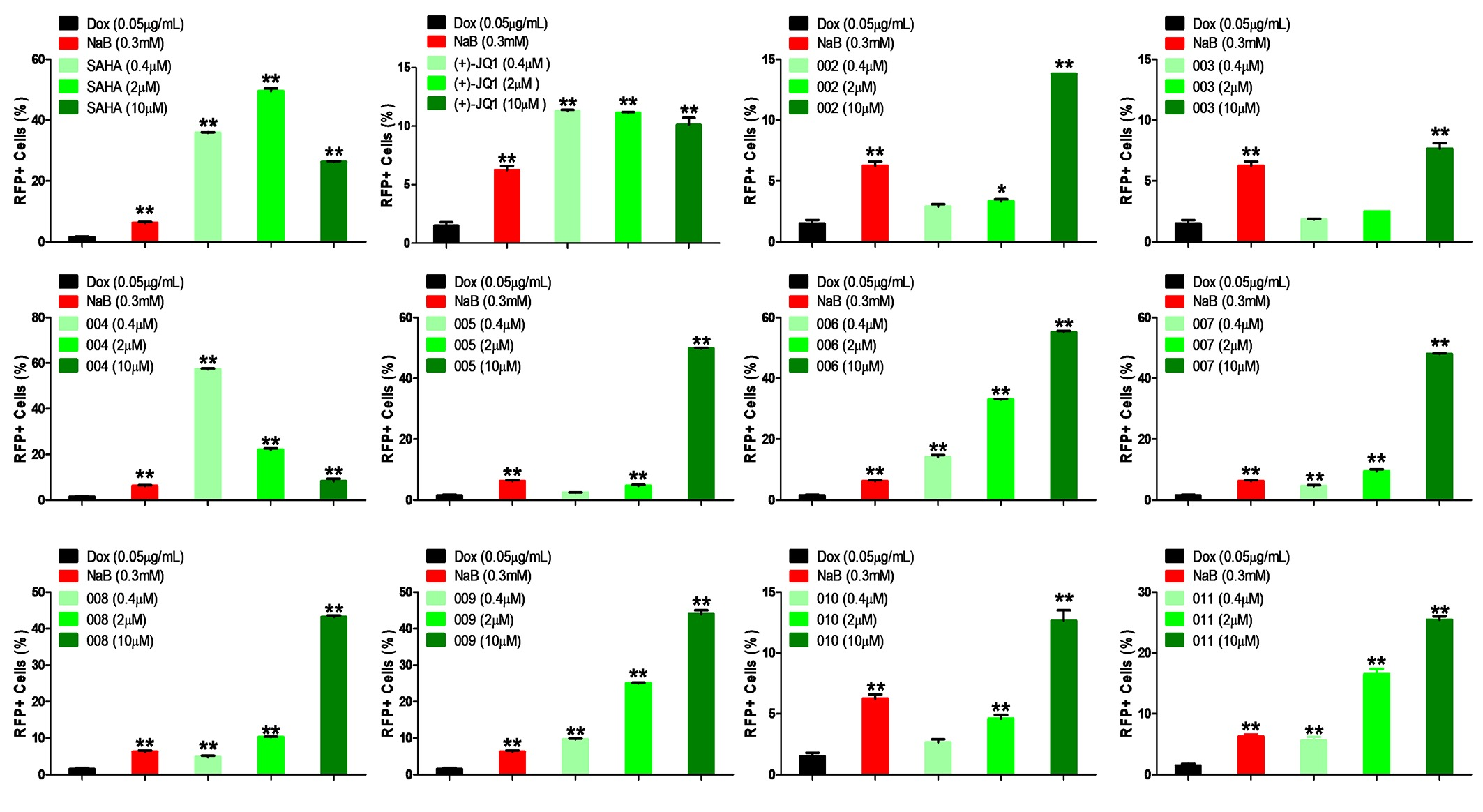

Supplement: S1 Fig — The iSLK.219 cells were treated with indicated concentrations of compounds with the addition of Dox (0.05 μg/mL) for 72 h, then the levels of RFP expressed cells were analyzed and quantified using flow cytometry. Error bars represent S.D. for 3 independent experiments, * = p<0.05, ** = p<0.01. (TIF) [file ppat.1011089.s002.tif]

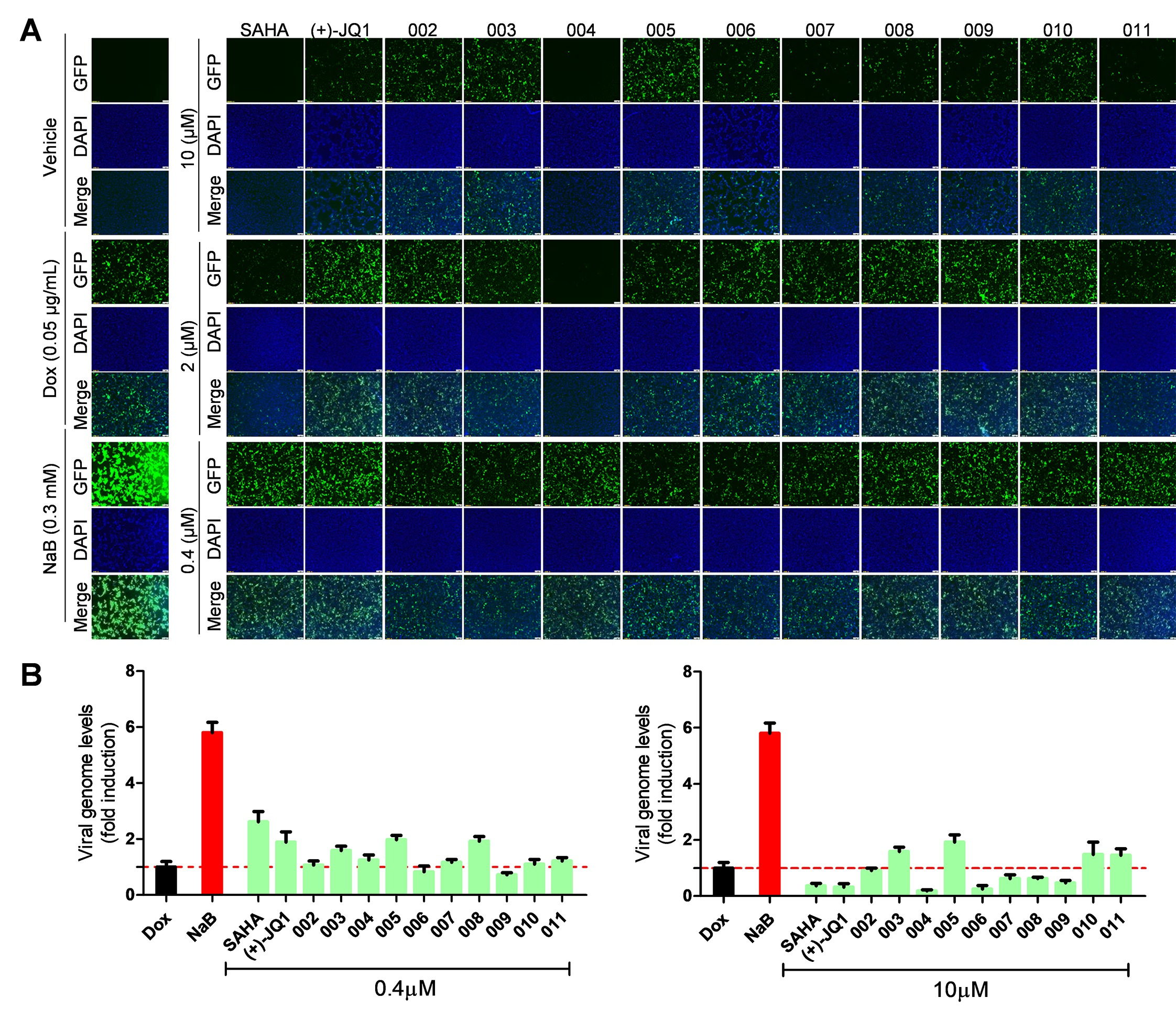

Supplement: S2 Fig — The iSLK.219 cells were treated by indicated concentrations of compounds with the addition of Dox (0.05 μg/mL) for 72 h treatment, then the supernatants were collected to infect HEK293T cells. (A) Cells were stained with DAPI at 48 h post-infection and the fluorescence signals were examined using fluorescence microscopy. (B) The viral DNA levels were quantified using qPCR as described in the Methods. Data was normalized as the fold change compared to the Dox control. (TIF) [file ppat.1011089.s003.tif]

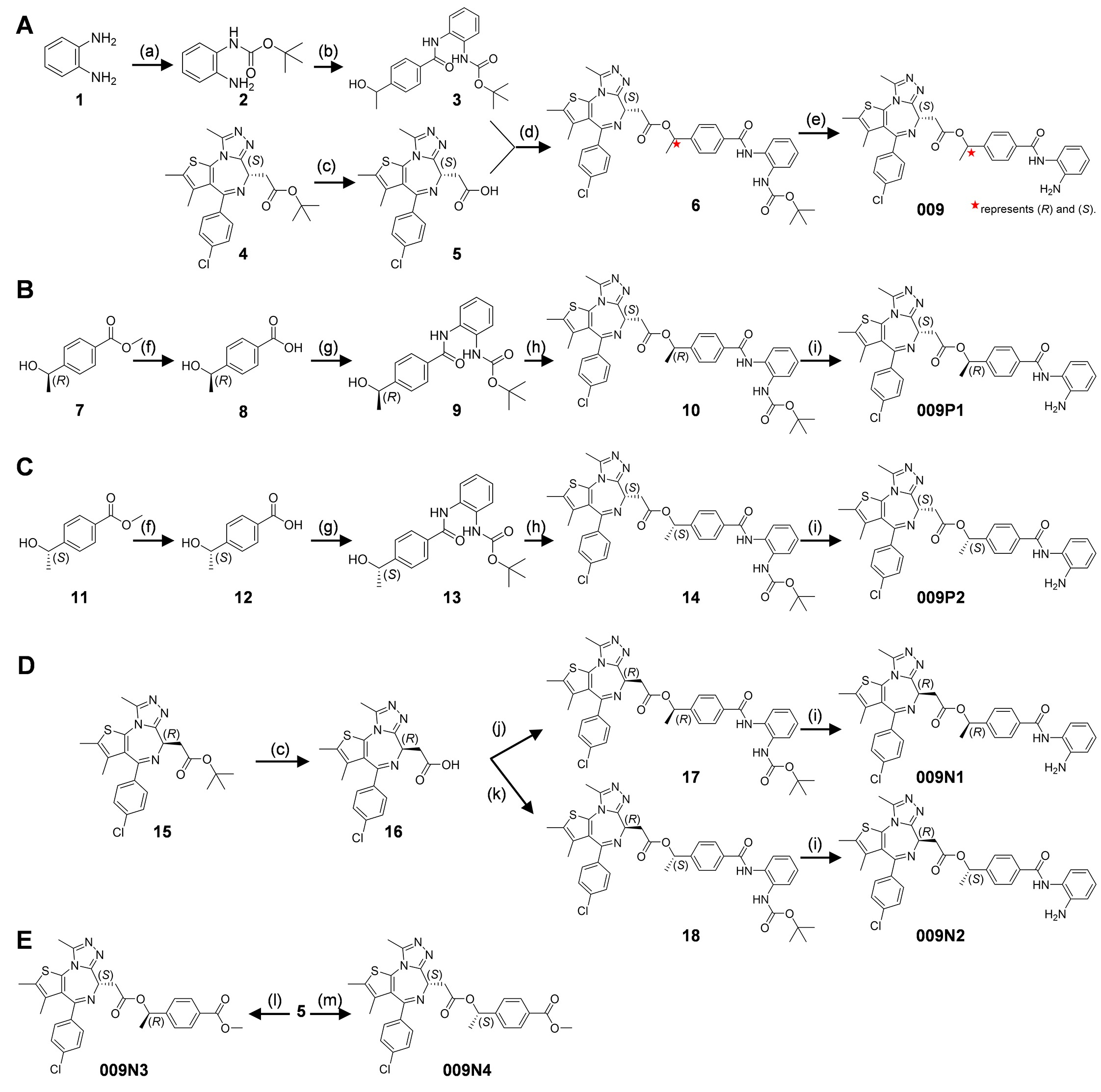

Supplement: S3 Fig — Synthesis of 009 (A), 009P1 (B), 009P2 (C), 009N1/009N2 (D) and 009N3/009N4 (E). Reagents and conditions: (a) (Boc)2O, NaOH, 1,4-dioxane, H2O, 0°C to rt, overnight; (b) 4-(1-hydroxyethyl)benzoic acid, HATU, DIPEA, DMF, 0°C to rt, overnight; (c) TFA, DCM, rt, overnight; (d) PyBOP, DIPEA, DMF, 0°C to rt, overnight; (e) TFA, DCM, 0°C to rt, 3 h; (f) LiOH·H2O, THF, MeOH, H2O, 0°C to rt, overnight; (g) 2, HATU, DIPEA, DMF, 0°C to rt, overnight; (h) 5, PyBOP, DIPEA, DMF, 0°C to rt, overnight; (i) TFA, DCM, 0°C to rt, 2-3 h; (j) 9, PyBOP, DIPEA, DMF, 0°C to rt, overnight; (k) 13, PyBOP, DIPEA, DMF, 0°C to rt, overnight; (l) 7, PyBOP, DIPEA, DMF, 0°C to rt, overnight; (m) 11, PyBOP, DIPEA, DMF, 0°C to rt, overnight. (TIF) [file ppat.1011089.s004.tif]

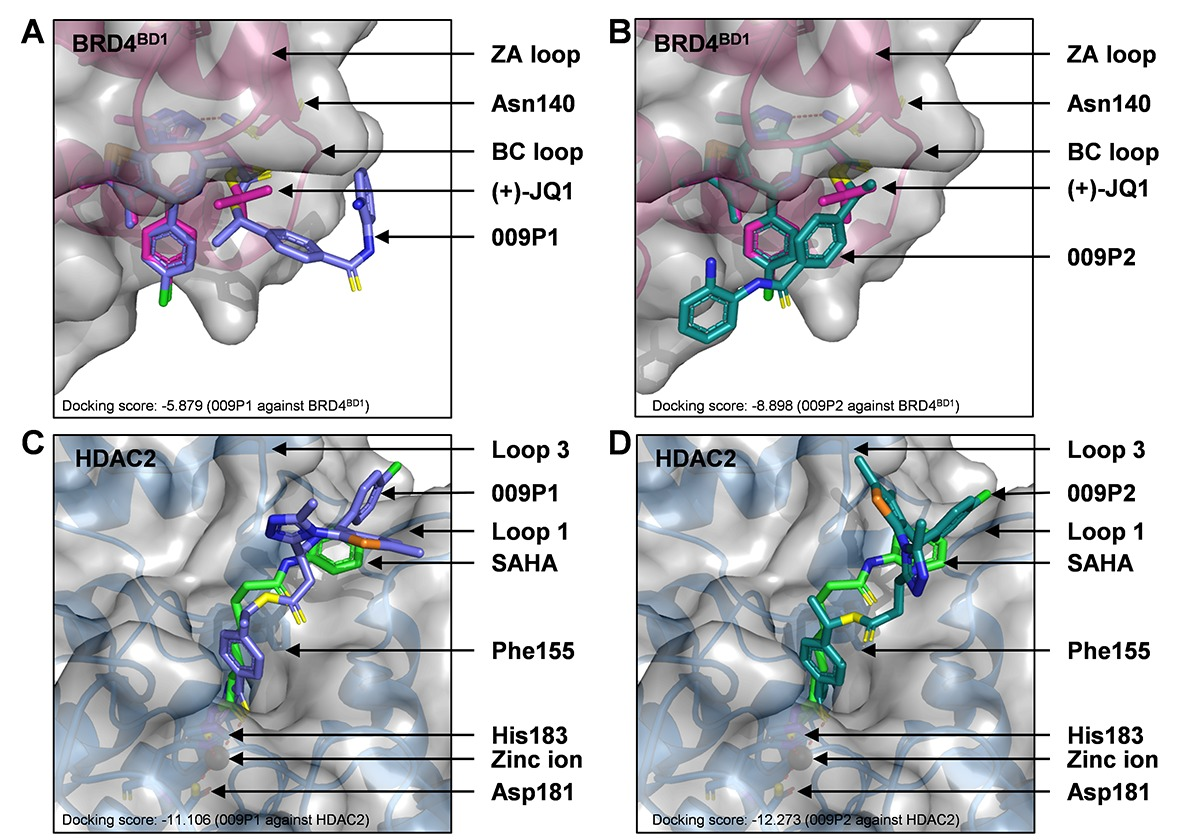

Supplement: S4 Fig — (A) The binding mode of 009P1 and (+)-JQ1 with BRD4BD1. BRD4BD1 is shown as hot pink cartoon with gray surface. Asn140 in BRD4BD1 is shown as sticks and colored in following pattern: atom C hot pink, atom N dark blue, and atom O yellow. (+)-JQ1 is shown as sticks and colored in following pattern: atom C hot pink, atom N dark blue, atom O yellow, atom S golden, and atom Cl green. 009P1 is shown as sticks and colored in following pattern: atom C light purple, atom N dark blue, atom O yellow, atom S golden, and atom Cl green. (B) The binding mode of 009P2 and (+)-JQ1 with BRD4BD1. BRD4BD1 is shown as hot pink cartoon with gray surface. Asn140 in BRD4BD1 is shown as sticks and colored in following pattern: atom C hot pink, atom N dark blue, and atom O yellow. (+)-JQ1 is shown as sticks and colored in following pattern: atom C hot pink, atom N dark blue, atom O yellow, atom S golden, and atom Cl green. 009P2 is shown as sticks and colored in following pattern: atom C dark green, atom N dark blue, atom O yellow, atom S golden, and atom Cl green. (C) The binding mode of 009P1 and SAHA with HDAC2. HDAC2 is shown as blue cartoon with gray surface. Asp181, His183, and Phe155 in HDAC2 are shown as sticks and colored in following pattern: atom C blue, atom N dark blue, and atom O yellow. 009P1 is shown as sticks and colored in following pattern: atom C light purple, atom N dark blue, atom O yellow, atom S golden, and atom Cl green. The catalytic zinc ion in the active site of HDAC2 is shown as black sphere. SAHA is shown as sticks and colored in following pattern: atom C green, atom N dark blue, and atom O yellow. (D) The binding mode of 009P2 and vorinostat with HDAC2. HDAC2 is shown as blue cartoon with gray surface. Asp181, His183, and Phe155 in HDAC2 are shown as sticks and colored in following pattern: atom C blue, atom N dark blue, and atom O yellow. 009P2 is shown as sticks and colored in following pattern: atom C dark green, atom N dark blue, atom O yellow, atom S golde [file ppat.1011089.s005.tif]

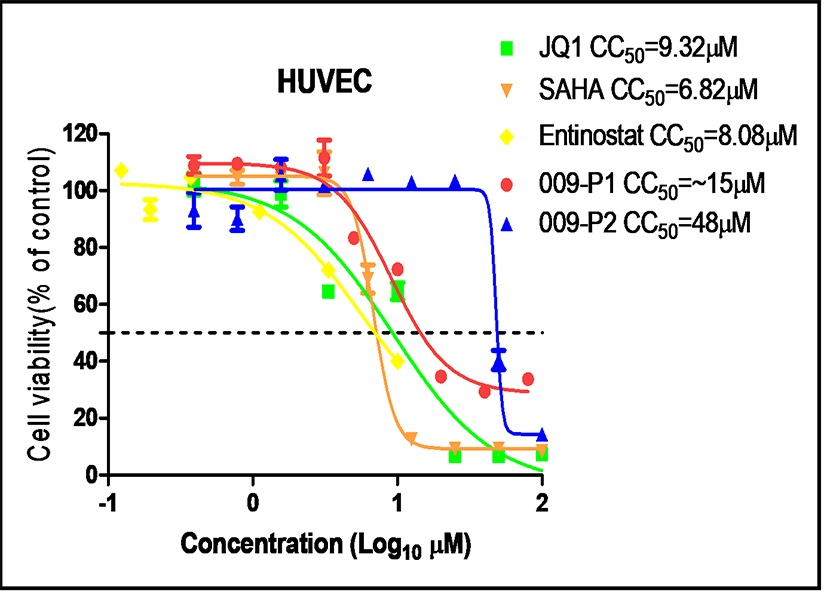

Supplement: S5 Fig — The primary cells HUVEC were treated with indicated concentrations of compounds for 72 h, then cell viability was assessed using the WST-1 assay. The CC50 for each compound was calculated from the dose-response curves using Graphpad Prism 5.0 software. Data was normalized as the fold change compared to the DMSO control. (TIF) [file ppat.1011089.s006.tif]

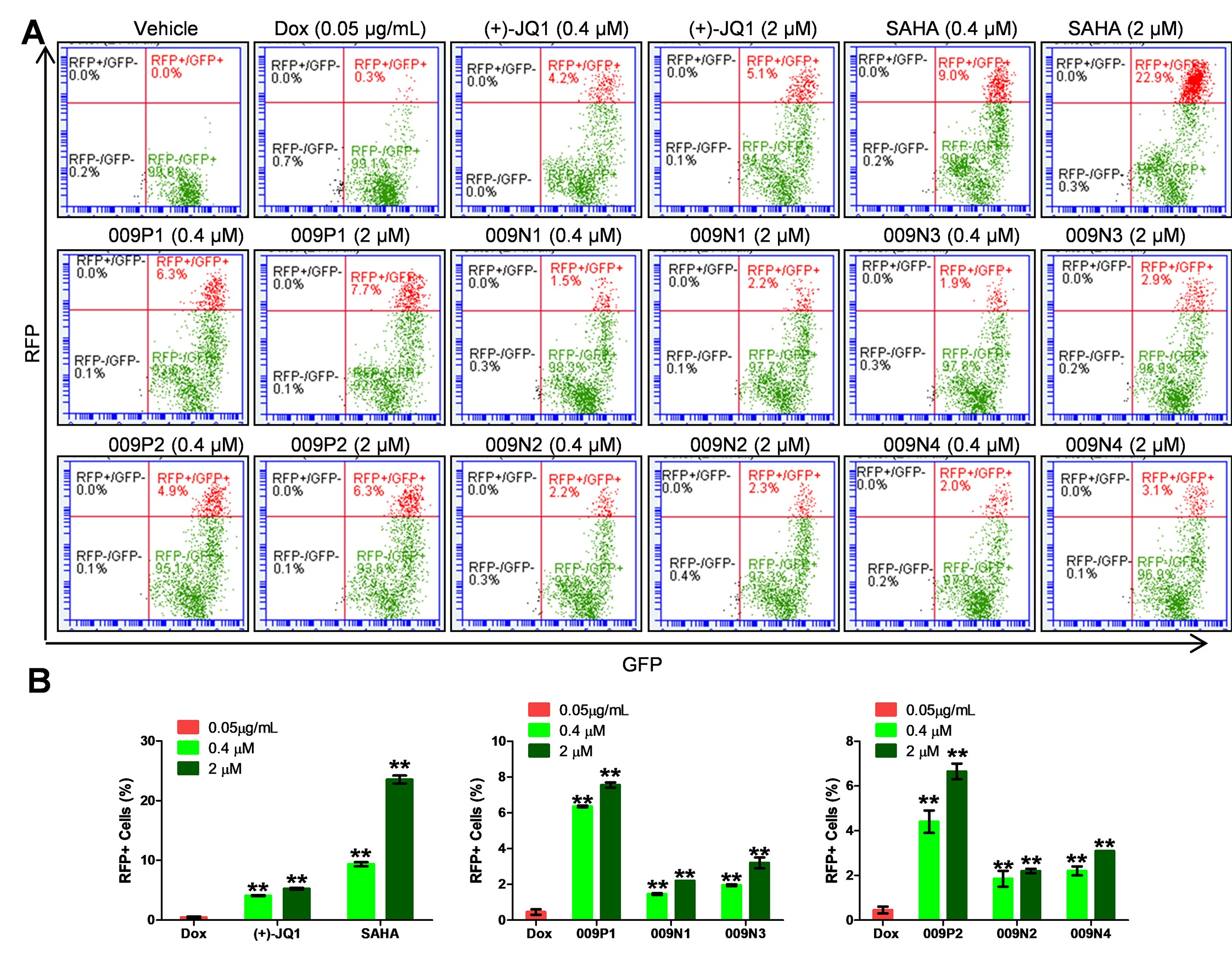

Supplement: S6 Fig — The iSLK.219 cells were treated with indicated concentrations of compounds in the addition to Dox (0.05 μg/mL) for 24 h, then, the levels of RFP expressed cells were examined (A) and quantified (B) using flow cytometer. Error bars represent S.D. for 3 independent experiments, ** = p<0.01. (TIF) [file ppat.1011089.s007.tif]

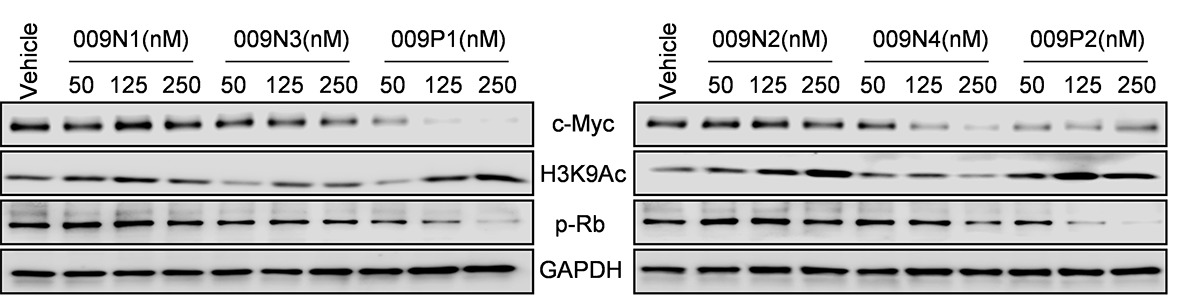

Supplement: S7 Fig — The BCBL-1 cells were treated with indicated concentrations of compounds for 48 h, then protein expression was determined using Western blot assays. (TIF) [file ppat.1011089.s008.tif]
