# Supplementary material for: Oncolytic strategy using new bifunctional HDACs/BRD4 inhibitors against virus-associated lymphomas
Source: PLoS Pathog. 2023 Jan 13;19(1):e1011089. doi: 10.1371/journal.ppat.1011089 (PMC9879403; doi:10.1371/journal.ppat.1011089)
Supplement: S1 Supplementary Methods — (DOCX) [file ppat.1011089.s009.docx]

**S1 Supplementary Methods: Supplementary methods about chemicals synthesis.**

**1. Synthesis of 1-(4-((2-aminophenyl)carbamoyl)phenyl)ethyl 2-((*S*)-4-(4-chlorophenyl)-2,3,9-trimethyl-6*H*-thieno[3,2-*f*][1,2,4]triazolo[4,3-*a*][1,4]diazepin-6-yl)acetate (009)**

**Synthesis of *tert*-butyl (2-aminophenyl)carbamate (2)**

To a mixture of *o*-phenylenediamine (**1**, 23.6 g, 220.0 mmol) and 1N sodium hydroxide aqueous solution (118.0 mL) in 1,4-dioxane (150.0 mL) was added a suspension of di-*tert*-butyl dicarbonate (52.5 g, 240.0 mmol) in 1,4-dioxane (100.0 mL) dropwise at 0 ℃. After addition, the reaction mixture was stirred at room temperature overnight. LC/MS analysis indicated the completed conversion. The reaction mixture was diluted with water (200.0 mL) and extracted with dichloromethane (200.0 mL * 3). The combined organic phases were dried over anhydrous sodium sulfate, filtered, concentrated under vacuum, absorbed onto silica gel, and purified via flash chromatography (ethyl acetate: hexanes = 1: 99 to 1: 9) to afford a light yellow solid as *tert*-butyl (2-aminophenyl)carbamate (**2**, 23.3 g, 51% yields). ^1^H NMR (400 MHz, DMSO-*d_6_*) δ 8.25 (s, 1H), 7.17 (d, *J* = 7.9 Hz, 1H), 6.83 (t, *J* = 7.6 Hz, 1H), 6.67 (d, *J* = 8.0 Hz, 1H), 6.51 (t, *J* = 7.6 Hz, 1H), 4.80 (s, 2H), 1.45 (s, 9H). ESI-MS *m/z*: 209 [M+H]^+^.

**Synthesis of *tert*-butyl (2-(4-(1-hydroxyethyl)benzamido)phenyl)carbamate (3)**

A mixture of 4-(1-hydroxyethyl)benzoic acid (0.2 g, 1.0 mmol) and *N*, *N*-diisopropylethylamine (0.6 g, 5.0 mmol) in anhydrous dimethylformamide (10.0 mL) was stirred at 0 ℃ for 5 minutes. *tert*-butyl (2-aminophenyl)carbamate (**2**, 0.2 g, 1.0 mmol) and 1-[bis(dimethylamino)methylene]-1*H*-1,2,3-triazolo[4,5-*b*]pyridinium 3-oxide hexafluorophosphate (0.5 g, 1.2 mmol) were successively added to the mixture at 0 ℃. After addition, the reaction mixture was stirred at room temperature overnight. LC/MS analysis indicated the completed conversion. The reaction mixture was diluted with iced water (20.0 mL) and extracted with dichloromethane (20.0 mL * 3). The combined organic phases were washed with brine (20 mL), dried over anhydrous sodium sulfate, filtered, concentrated under vacuum, absorbed onto silica gel, and purified via flash chromatography (ethyl acetate: hexanes = 1: 99 to 1: 1) to afford a light yellow foam as *tert*-butyl (2-(4-(1-hydroxyethyl)benzamido)phenyl)carbamate (**3**, 0.3 g, 71.5% yields). ^1^H NMR (400 MHz, DMSO-*d_6_*) 9.76 (s, 1H), 8.65 (s, 1H), 7.87 (d, *J* = 8.3 Hz, 2H), 7.54 – 7.48 (m, 2H), 7.46 (d, *J* = 8.3 Hz, 2H), 7.19 – 7.08 (m, 2H), 5.30 (d, *J* = 4.3 Hz, 1H), 4.82 – 4.74 (m, 1H), 1.41 (s, 9H), 1.31 (d, *J* = 6.5 Hz, 3H). ESI-MS *m/z*: 357 [M+H]^+^.

**Synthesis of (+)-JQ1 carboxylic acid (5)**

To a stirred solution of (+)-JQ1 (**4**, 1.0 g, 2.2 mmol) (MedChemExpress, catalogue number: HY-13030) in dichloromethane (15.0 mL) was added trifluoroacetic acid (5.0 mL). The reaction mixture was stirred at room temperature overnight. LC/MS analysis indicated the completed conversion. The reaction mixture was concentrated under vacuum, absorbed onto Celite and purified via C18 reversed flash chromatography (deionized water: methanol = 95: 5 to 100% methanol) to afford a white foam as (+)-JQ1 carboxylic acid (**5**, 0.7 g, 84.3% yields). ^1^H NMR (400 MHz, DMSO-*d_6_*) δ 7.45 (d, *J* = 8.7 Hz, 2H), 7.39 (d, *J* = 8.6 Hz, 2H), 4.40 (t, *J* = 7.1 Hz, 1H), 3.40 – 3.20 (m, 2H), 2.55 (s, 3H), 2.37 (s, 3H), 1.58 (s, 3H). ESI-MS *m/z*: 401 [M+H]^+^.

**Synthesis of 1-(4-((2-((tert-butoxycarbonyl)amino)phenyl)carbamoyl)phenyl)ethyl 2-((*S*)-4-(4-chlorophenyl)-2,3,9-trimethyl-6*H*-thieno[3,2-*f*][1,2,4]triazolo[4,3-*a*][1,4]diazepin-6-yl)acetate (6)**

To a solution of (+)-JQ1 carboxylic acid (**5**, 0.1 g, 0.3 mmol) and *N*, *N*-diisopropylethylamine (0.1 g, 0.9 mmol) in anhydrous dimethylformamide (5.0 mL) was added benzotriazol-1-yl-oxytripyrrolidinophosphonium hexafluorophosphate (0.2 g, 0.3 mmol) at 0 ℃. The resulting light-yellow solution was allowed to stir at 0 ℃ for 5 minutes under nitrogen atmosphere. Then *tert*-butyl (2-(4-(1-hydroxyethyl)benzamido)phenyl)carbamate (**3**, 0.1 g, 0.3 mmol) was added and the reaction mixture was stirred at room temperature overnight under nitrogen atmosphere. LC/MS analysis indicated the completed conversion. The resulting mixture was diluted with iced water (20.0 mL) and extracted with ethyl acetate (20.0 mL * 3). The combined organic phases were washed with brine (20 mL), dried over anhydrous sodium, filtered, concentrated under vacuum, absorbed onto silica gel, and purified via C18 reversed flash chromatography (methanol : water = 5 : 95 to 100% methanol) to afford a beige foam as 1-(4-((2-((tert-butoxycarbonyl)amino)phenyl)carbamoyl)phenyl)ethyl 2-((*S*)-4-(4-chlorophenyl)-2,3,9-trimethyl-6*H*-thieno[3,2-*f*][1,2,4]triazolo[4,3-*a*][1,4]diazepin-6-yl)acetate (**6**, 84.0 mg, 37.9% yields). ^1^H NMR (400 MHz, DMSO-*d_6_*) 9.90 – 9.77 (m, 1H), 8.61 (s, 1H), 7.90 (dd, *J* = 23.3, 8.2 Hz, 2H), 7.56 – 7.46 (m, 4H), 7.46 – 7.37 (m, 2H), 7.28 (dd, *J* = 8.5, 2.3 Hz, 2H), 7.21 – 7.07 (m, 2H), 5.97 – 5.87 (m, 1H), 4.51 – 4.44 (m, 1H), 3.47 (d, *J* = 7.2 Hz, 2H), 2.61 – 2.54 (m, 3H), 2.37 (s, 3H), 1.58 – 1.53 (m, 3H), 1.50 (d, *J* = 6.6 Hz, 3H), 1.43 – 1.34 (m, 9H). ESI-MS *m/z*: 739 [M+H]^+^.

**Synthesis of 1-(4-((2-aminophenyl)carbamoyl)phenyl)ethyl 2-((*S*)-4-(4-chlorophenyl)-2,3,9-trimethyl-6*H*-thieno[3,2-*f*][1,2,4]triazolo[4,3-*a*][1,4]diazepin-6-yl)acetate (009)**

To a stirred solution of 1-(4-((2-((tert-butoxycarbonyl)amino)phenyl)carbamoyl)phenyl)ethyl 2-((*S*)-4-(4-chlorophenyl)-2,3,9-trimethyl-6*H*-thieno[3,2-*f*][1,2,4]triazolo[4,3-*a*][1,4]diazepin-6-yl)acetate (**6**, 10.0 mg, 0.014 mmol) in dichloromethane (2.0 mL) was added trifluoroacetic acid (0.5 mL). The reaction mixture was stirred at room temperature for 3 hours. LC/MS analysis indicated the completed conversion, and the resulting mixture was diluted with 5.0 mL of dichloromethane. The mixture was basified via the addition of saturated sodium carbonate aqueous solution to pH = 10 and the resulting mixture was stirred at room temperature for 5 minutes. Then the mixture was extracted with dichloromethane (20.0 mL * 3). The combined organic phases were washed with brine, dried over anhydrous sodium sulfate, filtered, concentrated under vacuum, absorbed onto silica gel, and purified via flash chromatography (dichloromethane: methanol = 99: 1 to 95: 5) to afford a light-yellow solid as 1-(4-((2-aminophenyl)carbamoyl)phenyl)ethyl 2-((*S*)-4-(4-chlorophenyl)-2,3,9-trimethyl-6*H*-thieno[3,2-*f*][1,2,4]triazolo[4,3-*a*][1,4]diazepin-6-yl)acetate (**009**, 7.4 mg, 82.8% yields). ^1^H NMR (400 MHz, DMSO-*d_6_*) 9.65 (d, *J* = 13.9 Hz, 1H), 7.93 (dd, *J* = 24.1, 8.1 Hz, 2H), 7.53 – 7.46 (m, 2H), 7.43 (d, *J* = 8.7 Hz, 2H), 7.28 (d, *J* = 8.6 Hz, 2H), 7.13 (t, *J* = 8.2 Hz, 1H), 6.98 – 6.90 (m, 1H), 6.78 – 6.72 (m, 1H), 6.61 – 6.53 (m, 1H), 5.93 (q, *J* = 6.5 Hz, 1H), 4.88 (s, 2H), 4.52 – 4.42 (m, 1H), 3.50 – 3.44 (m, 2H), 2.61 – 2.52 (m, 3H), 2.37 (s, 3H), 1.60 – 1.54 (m, 3H), 1.50 (d, *J* = 6.6 Hz, 3H). ^13^C NMR (101 MHz, DMSO-*d_6_*) δ 170.30, 170.20, 165.39, 165.31, 163.79, 163.66, 155.00, 154.98, 150.40, 150.33, 145.51, 145.31, 143.64, 143.60, 136.96, 135.79, 135.75, 134.38, 134.37, 132.80, 132.76, 131.21, 131.15, 130.57 (2C), 130.52 (2C), 130.31, 130.24, 129.85, 129.83, 128.92 (2C), 128.88 (2C), 128.38 (2C), 128.36 (2C), 127.20, 127.17, 126.99, 126.95, 126.13 (2C), 126.06 (2C), 123.63, 116.64, 116.53, 116.51, 72.09, 72.06, 53.97, 53.88, 37.20, 37.04, 22.58, 22.54, 14.47, 14.44, 13.12, 11.74, 11.72. ESI-MS *m/z*: 639 [M+H]^+^.

**2. Synthesis of (*R*)-1-(4-((2-aminophenyl)carbamoyl)phenyl)ethyl 2-((*S*)-4-(4-chlorophenyl)-2,3,9-trimethyl-6*H*-thieno[3,2-*f*][1,2,4]triazolo[4,3-*a*][1,4]diazepin-6-yl)acetate (009P1)**

**Synthesis of (*R*)-4-(1-hydroxyethyl)benzoic acid (8)**

To a solution of methyl (*R*)-4-(1-hydroxyethyl)benzoate (**7**, 2.0 g, 11.1 mmol) (Millipore Sigma, product number: ENA424046216) in tetrahydrofuran (42.0 mL) and menthol (42.0 mL) was added a solution of lithium hydroxide monohydrate (1.4 g, 33.3 mmol) in water (14.0 mL) dropwise at 0 ℃. After addition, the reaction mixture was stirred at room temperature overnight. LC/MS analysis indicated the completed conversion. The reaction mixture was concentrated under vacuum to remove the organic solvents. The residue was diluted with water (10.0 mL) and adjusted to pH 4~5 via the addition of 1N hydrochloric acid aqueous solution dropwise at 0 ℃ to generate a white suspension. After filtration, the collected solid was washed with iced water (10.0 mL) and dried under vacuum to afford a white solid as (*R*)-4-(1-hydroxyethyl)benzoic acid (**8**, 1.7 g, 92.5% yields). ^1^H NMR (400 MHz, DMSO-*d_6_*) δ 12.76 (s, 1H), 7.85 (d, *J* = 8.3 Hz, 2H), 7.41 (d, *J* = 8.2 Hz, 2H), 5.26 (d, *J* = 4.2 Hz, 1H), 4.74 (qd, *J* = 6.5, 4.1 Hz, 1H), 1.28 (d, *J* = 6.5 Hz, 3H). ESI-MS *m/z*: 167 [M+H]^+^.

**Synthesis of *tert*-butyl (*R*)-(2-(4-(1-hydroxyethyl)benzamido)phenyl)carbamate (9)**

A mixture of (*R*)-4-(1-hydroxyethyl)benzoic acid (**8**, 0.6 g, 3.6 mmol) and *N*, *N*-diisopropylethylamine (3.2 mL, 18.0 mmol) in anhydrous dimethylformamide (50.0 mL) was stirred at 0 ℃ for 5 minutes. *tert*-butyl (2-aminophenyl)carbamate (**2**, 0.8 g, 3.6 mmol) and 1-[bis(dimethylamino)methylene]-1*H*-1,2,3-triazolo[4,5-*b*]pyridinium 3-oxide hexafluorophosphate (1.6 g, 4.3 mmol) were successively added to the mixture at 0 ℃. After addition, the reaction mixture was stirred at room temperature overnight. LC/MS analysis indicated the completed conversion. The reaction mixture was diluted with iced water (200.0 mL) and extracted with dichloromethane (200.0 mL * 3). The combined organic phases were washed with brine (200 mL), dried over anhydrous sodium sulfate, filtered, concentrated under vacuum, absorbed onto silica gel, and purified via flash chromatography (ethyl acetate: hexanes = 1: 99 to 1: 1) to afford a light yellow foam as *tert*-butyl (*R*)-(2-(4-(1-hydroxyethyl)benzamido)phenyl)carbamate (**9**, 1.2 g, 96.4% yields). ^1^H NMR (400 MHz, DMSO-*d_6_*) δ 9.75 (s, 1H), 8.63 (s, 1H), 7.87 (d, *J* = 8.3 Hz, 2H), 7.54 – 7.47 (m, 2H), 7.46 (d, *J* = 8.3 Hz, 2H), 7.20 – 7.09 (m, 2H), 5.28 (d, *J* = 4.3 Hz, 1H), 4.81 – 4.74 (m, 1H), 1.41 (s, 9H), 1.31 (d, *J* = 6.5 Hz, 3H). ESI-MS *m/z*: 379 [M+Na]^+^.

**Synthesis of (*R*)-1-(4-((2-((*tert*-butoxycarbonyl)amino)phenyl)carbamoyl)phenyl)ethyl 2-((*S*)-4-(4-chlorophenyl)-2,3,9-trimethyl-6*H*-thieno[3,2-*f*][1,2,4]triazolo[4,3-*a*][1,4]diazepin-6-yl)acetate (10)**

To a solution of (+)-JQ1 carboxylic acid (**5**, 2.9 g, 7.2 mmol), benzotriazol-1-yl-oxytripyrrolidinophosphonium hexafluorophosphate (4.5 g, 8.7 mmol) and *N*, *N*-diisopropylethylamine (3.6 mL, 21.7 mmol) in anhydrous dimethylformamide (20.0 mL) was added *tert*-butyl (*R*)-(2-(4-(1-hydroxyethyl)benzamido)phenyl)carbamate (**9**, 2.8 g, 8.0 mmol) at 0 ℃. After addition, the reaction mixture was stirred at room temperature overnight. LC/MS analysis indicated the completed conversion. The resulting mixture was diluted with water (200.0 mL) and extracted with dichloromethane (200.0 mL * 3). The combined organic phases were washed with brine, dried over anhydrous sodium sulfate, filtered, concentrated under vacuum, absorbed onto silica gel, and purified via flash chromatography (ethyl acetate: hexanes = 1: 99 to 100% ethyl acetate) to afford a white form as (*R*)-1-(4-((2-((*tert*-butoxycarbonyl)amino)phenyl)carbamoyl)phenyl)ethyl 2-((*S*)-4-(4-chlorophenyl)-2,3,9-trimethyl-6*H*-thieno[3,2-*f*][1,2,4]triazolo[4,3-*a*][1,4]diazepin-6-yl)acetate (**10**, 3.3 g, 61.7 yields). ^1^H NMR (400 MHz, DMSO-*d_6_*) δ 9.84 (s, 1H), 8.61 (s, 1H), 7.93 (d, *J* = 8.3 Hz, 2H), 7.52 (t, *J* = 9.0 Hz, 4H), 7.43 (d, *J* = 8.8 Hz, 2H), 7.28 (d, *J* = 8.4 Hz, 2H), 7.15 (dtd, *J* = 22.4, 7.5, 1.7 Hz, 2H), 5.93 (q, *J* = 6.5 Hz, 1H), 4.47 (t, *J* = 7.3 Hz, 1H), 3.47 (d, *J* = 7.3 Hz, 2H), 2.57 (s, 3H), 2.40 – 2.34 (m, 3H), 1.56 (s, 3H), 1.50 (d, *J* = 6.6 Hz, 3H), 1.39 (s, 9H). ESI-MS *m/z*: 739 [M+H]^+^.

**Synthesis of (*R*)-1-(4-((2-aminophenyl)carbamoyl)phenyl)ethyl 2-((*S*)-4-(4-chlorophenyl)-2,3,9-trimethyl-6*H*-thieno[3,2-*f*][1,2,4]triazolo[4,3-*a*][1,4]diazepin-6-yl)acetate (009P1)**

To a stirred solution of (*R*)-1-(4-((2-((*tert*-butoxycarbonyl)amino)phenyl)carbamoyl)phenyl)ethyl 2-((*S*)-4-(4-chlorophenyl)-2,3,9-trimethyl-6*H*-thieno[3,2-*f*][1,2,4]triazolo[4,3-*a*][1,4]diazepin-6-yl)acetate (**10**, 1.0 g, 1.4 mmol) in dichloromethane (20.0 mL) was added trifluoroacetic acid (5.2 mL). The reaction mixture was stirred at room temperature for 3 hours. LC/MS analysis indicated the completed conversion, and the resulting mixture was concentrated under vacuum to afford a light-yellow oil, which was diluted with 5.0 mL of dichloromethane. The mixture was basified via the addition of saturated sodium carbonate aqueous solution to pH = 10 and the resulting mixture was stirred at room temperature for 5 minutes. Then the mixture was extracted with dichloromethane (20.0 mL * 3). The combined organic phases were washed with brine, dried over anhydrous sodium sulfate, filtered, concentrated under vacuum, absorbed onto silica gel, and purified via flash chromatography (ethyl acetate: methanol = 1: 99 to 3: 97) to afford a beige foam as (*R*)-1-(4-((2-aminophenyl)carbamoyl)phenyl)ethyl 2-((*S*)-4-(4-chlorophenyl)-2,3,9-trimethyl-6*H*-thieno[3,2-*f*][1,2,4]triazolo[4,3-*a*][1,4]diazepin-6-yl)acetate (**009P1**, 0.9 g, 98.5% yields). ^1^H NMR (400 MHz, DMSO-*d_6_*) δ 9.67 (s, 1H), 7.96 (d, *J* = 8.2 Hz, 2H), 7.48 (d, *J* = 8.3 Hz, 2H), 7.44 (d, *J* = 8.6 Hz, 2H), 7.28 (d, *J* = 8.5 Hz, 2H), 7.15 (d, *J* = 6.7 Hz, 1H), 6.99 – 6.92 (m, 1H), 6.76 (dd, *J* = 8.0, 1.4 Hz, 1H), 6.58 (t, *J* = 7.5 Hz, 1H), 5.93 (q, *J* = 6.5 Hz, 1H), 4.88 (s, 2H), 4.47 (t, *J* = 7.3 Hz, 1H), 3.47 (d, *J* = 7.3 Hz, 2H), 2.57 (s, 3H), 2.37 (s, 3H), 1.56 (d, *J* = 0.9 Hz, 3H), 1.50 (d, *J* = 6.6 Hz, 3H). ^13^C NMR (101 MHz, DMSO-*d_6_*) δ 170.31, 165.39, 163.66, 155.00, 150.33, 145.51, 143.63, 136.96, 135.79, 134.36, 132.80, 131.21, 130.52 (2C), 130.24, 129.83, 128.92 (2C), 128.36 (2C), 127.19, 126.99, 126.06 (2C), 123.64, 116.66, 116.55, 72.06, 53.97, 37.04, 22.58, 14.47, 13.12, 11.72. ESI-MS *m/z*: 639 [M+H]^+^. HRMS (ESI^+^) *m/z* calcd for C_34_H_31_ClN_6_O_3_S: 638.1867; found 639.1915 [M+H]^+^. HPLC purity: 98.36%.

**3. Synthesis of of (*S*)-1-(4-((2-aminophenyl)carbamoyl)phenyl)ethyl 2-((*S*)-4-(4-chlorophenyl)-2,3,9-trimethyl-6*H*-thieno[3,2-*f*][1,2,4]triazolo[4,3-*a*][1,4]diazepin-6-yl)acetate (009P2)**

**Synthesis of (*S*)-4-(1-hydroxyethyl)benzoic acid (12)**

To a solution of methyl (*S*)-4-(1-hydroxyethyl)benzoate (**11**, 2.0g, 11.1 mmol) (Millipore Sigma, product number: ENA245487789) in tetrahydrofuran (42.0 mL) and menthol (42.0 mL) was added a solution of lithium hydroxide monohydrate (1.4 g, 33.3 mmol) in water (14.0 mL) dropwise at 0 ℃. After addition, the reaction mixture was stirred at room temperature overnight. LC/MS analysis indicated the completed conversion. The reaction mixture was concentrated under vacuum to remove the organic solvents. The residue was diluted with water (20.0 mL) and adjusted to pH 4~5 via the addition of 1N hydrochloric acid aqueous solution dropwise at 0 ℃ to generate a white suspension. After filtration, the collected solid was washed with iced water (20.0 mL) and dried under vacuum to afford a white solid as (*S*)-4-(1-hydroxyethyl)benzoic acid (**12**, 1.6 g, 84.0% yields). ^1^H NMR (400 MHz, DMSO-*d_6_*) δ 12.76 (s, 1H), 7.85 (d, *J* = 8.3 Hz, 2H), 7.42 (d, *J* = 8.2 Hz, 2H), 5.26 (d, *J* = 4.3 Hz, 1H), 4.84 – 4.64 (m, 1H), 1.29 (d, *J* = 6.5 Hz, 3H). ESI-MS *m/z*: 189 [M+Na]^+^.

**Synthesis of *tert*-butyl (*S*)-(2-(4-(1-hydroxyethyl)benzamido)phenyl)carbamate (13)**

A mixture of (*S*)-4-(1-hydroxyethyl)benzoic acid (**12**, 0.7 g, 4.2 mmol) and *N*, *N*-diisopropylethylamine (3.7 mL, 21.0 mmol) in anhydrous dimethylformamide (20.0 mL) was stirred at 0 ℃ for 5 minutes. *tert*-butyl (2-aminophenyl)carbamate (**2**, 0.9 g, 4.2 mmol) and 1-[bis(dimethylamino)methylene]-1*H*-1,2,3-triazolo[4,5-*b*]pyridinium 3-oxide hexafluorophosphate (1.9 g, 5.0 mmol) were successively added to the mixture at 0 ℃. After addition, the reaction mixture was stirred at room temperature overnight. LC/MS analysis indicated the completed conversion. The reaction mixture was diluted with iced water (50.0 mL) and extracted with dichloromethane (50.0 mL * 3). The combined organic phases were washed with brine, dried over anhydrous sodium sulfate, filtered, concentrated under vacuum, absorbed onto silica gel, and purified via flash chromatography (ethyl acetate: hexanes = 1: 99 to 1: 1) to afford a white foam as *tert*-butyl (*S*)-(2-(4-(1-hydroxyethyl)benzamido)phenyl)carbamate (**13**, 1.2 g, 80.0% yields). ^1^H NMR (400 MHz, DMSO-*d_6_*) δ 9.76 (s, 1H), 8.66 (s, 1H), 7.87 (d, *J* = 8.3 Hz, 2H), 7.51 (ddd, *J* = 9.6, 7.8, 1.8 Hz, 2H), 7.46 (d, *J* = 8.3 Hz, 2H), 7.19 – 7.08 (m, 2H), 5.30 (d, *J* = 4.3 Hz, 1H), 4.83 – 4.72 (m, 1H), 1.41 (s, 9H), 1.31 (d, *J* = 6.4 Hz, 3H). ESI-MS *m/z*: 357 [M+H]^+^.

**Synthesis of (*S*)-1-(4-((2-((*tert*-butoxycarbonyl)amino)phenyl)carbamoyl)phenyl)ethyl 2-((*S*)-4-(4-chlorophenyl)-2,3,9-trimethyl-6*H*-thieno[3,2-*f*][1,2,4]triazolo[4,3-*a*][1,4]diazepin-6-yl)acetate (14)**

To a solution of (+)-JQ1 carboxylic acid (**5**, 1.4 g, 3.6 mmol), benzotriazol-1-yl-oxytripyrrolidinophosphonium hexafluorophosphate (2.2 g, 4.3 mmol) and *N*, *N*-diisopropylethylamine (1.3 g, 10.7 mmol) in anhydrous dimethylformamide (35.0 mL) was added *tert*-butyl (*S*)-(2-(4-(1-hydroxyethyl)benzamido)phenyl)carbamate (**13**, 1.4 g, 3.9 mmol) at 0 ℃. After addition, the reaction mixture was stirred at room temperature overnight. LC/MS analysis indicated the completed conversion. The resulting mixture was diluted with water (50 mL) and extracted with dichloromethane (50 mL * 3). The combined organic phases were washed with brine, dried over anhydrous sodium sulfate, filtered, concentrated under vacuum, absorbed onto silica gel, and purified via flash chromatography (ethyl acetate: hexanes = 1: 99 to 100% ethyl acetate) to afford a white form as (*S*)-1-(4-((2-((*tert*-butoxycarbonyl)amino)phenyl)carbamoyl)phenyl)ethyl 2-((*S*)-4-(4-chlorophenyl)-2,3,9-trimethyl-6*H*-thieno[3,2-*f*][1,2,4]triazolo[4,3-*a*][1,4]diazepin-6-yl)acetate (**14**, 1.4 g, 53.0% yields). ^1^H NMR (400 MHz, DMSO-*d_6_*) δ 9.83 (s, 1H), 8.60 (s, 1H), 7.90 (d, *J* = 8.1 Hz, 2H), 7.54 (d, *J* = 8.3 Hz, 2H), 7.51 (d, *J* = 7.8 Hz, 1H), 7.45 (d, *J* = 8.5 Hz, 2H), 7.31 (d, *J* = 8.5 Hz, 2H), 7.20 (t, *J* = 7.7 Hz, 1H), 7.14 (t, *J* = 7.9 Hz, 1H), 5.96 (q, *J* = 6.6 Hz, 1H), 4.54 – 4.47 (m, 1H), 3.51 (s, 2H), 2.59 (s, 3H), 2.40 (s, 3H), 1.59 (s, 3H), 1.53 (d, *J* = 6.6 Hz, 3H), 1.42 (s, 9H). ESI-MS *m/z*: 739 [M+H]^+^.

**Synthesis of (*S*)-1-(4-((2-aminophenyl)carbamoyl)phenyl)ethyl 2-((*S*)-4-(4-chlorophenyl)-2,3,9-trimethyl-6*H*-thieno[3,2-*f*][1,2,4]triazolo[4,3-*a*][1,4]diazepin-6-yl)acetate (009P2)**

To a stirred solution of (*S*)-1-(4-((2-((*tert*-butoxycarbonyl)amino)phenyl)carbamoyl)phenyl)ethyl 2-((*S*)-4-(4-chlorophenyl)-2,3,9-trimethyl-6*H*-thieno[3,2-*f*][1,2,4]triazolo[4,3-*a*][1,4]diazepin-6-yl)acetate (**14**, 0.8 g, 1.1 mmol) in dichloromethane (20.0 mL) was added trifluoroacetic acid (4.0 mL). The reaction mixture was stirred at 0 ^o^C for 3 hours. LC/MS analysis indicated the completed conversion, and the resulting mixture was concentrated under vacuum to afford a light-yellow oil, which was diluted with 5.0 mL of dichloromethane. The mixture was basified via the addition of saturated sodium carbonate aqueous solution to pH = 10 and the resulting mixture was stirred at room temperature for 5 minutes. Then the mixture was extracted with dichloromethane (20.0 mL * 3). The combined organic phases were washed with brine, dried over anhydrous sodium sulfate, filtered, concentrated under vacuum, absorbed onto silica gel, and purified via flash chromatography (ethyl acetate: methanol = 1: 99 to 3: 97) to afford a beige foam as (*S*)-1-(4-((2-aminophenyl)carbamoyl)phenyl)ethyl 2-((*S*)-4-(4-chlorophenyl)-2,3,9-trimethyl-6*H*-thieno[3,2-*f*][1,2,4]triazolo[4,3-*a*][1,4]diazepin-6-yl)acetate (**009P2**, 0.6 g, 92.9% yields). ^1^H NMR (400 MHz, DMSO-*d_6_*) δ 9.63 (s, 1H), 7.90 (d, *J* = 8.2 Hz, 2H), 7.49 (d, *J* = 8.3 Hz, 2H), 7.44 (d, *J* = 8.6 Hz, 2H), 7.28 (d, *J* = 8.6 Hz, 2H), 7.12 (d, *J* = 7.2 Hz, 1H), 6.98 – 6.91 (m, 1H), 6.75 (dd, *J* = 8.0, 1.4 Hz, 1H), 6.57 (td, *J* = 7.5, 1.4 Hz, 1H), 5.93 (q, *J* = 6.5 Hz, 1H), 4.86 (s, 2H), 4.51 – 4.46 (m, 1H), 3.51 – 3.44 (m, 2H), 2.57 (s, 3H), 2.38 (s, 3H), 1.57 (s, 3H), 1.50 (d, *J* = 6.6 Hz, 3H). ^13^C NMR (101 MHz, DMSO-*d_6_*) δ 170.20, 165.32, 163.80, 154.98, 150.41, 145.32, 143.60, 136.97, 135.76, 134.38, 132.77, 131.17, 130.58 (2C), 130.32, 129.86, 128.89 (2C), 128.38 (2C), 127.17, 126.96, 126.13 (2C), 123.64, 116.64, 116.52, 72.12, 53.89, 37.21, 22.54, 14.45, 13.11, 11.72. ESI-MS *m/z*: 639 [M+H]^+^. HRMS (ESI^+^) *m/z* calcd for C_34_H_31_ClN_6_O_3_S: 638.1867; found 639.1924 [M+H]^+^. HPLC purity: 95.62%.

**4. Synthesis of (*R*)-1-(4-((2-aminophenyl)carbamoyl)phenyl)ethyl 2-((*R*)-4-(4-chlorophenyl)-2,3,9-trimethyl-6*H*-thieno[3,2-*f*][1,2,4]triazolo[4,3-*a*][1,4]diazepin-6-yl)acetate (009N1)**

**Synthesis of (-)-JQ1 carboxylic acid (16)**

To a stirred solution of (-)-JQ1 (**15**, 90.0 mg, 0.2 mmol) (Combi-Blocks, catalog number: QJ-8217) in dichloromethane (4.0 mL) was added trifluoroacetic acid (0.5 mL). The reaction mixture was stirred at room temperature overnight. LC/MS analysis indicated the completed conversion. The reaction mixture was concentrated under vacuum, absorbed onto Celite and purified via C18 reversed flash chromatography (deionized water: methanol = 95: 5 to 100% methanol) to afford a white foam as (-)-JQ1 carboxylic acid (**16**, 78.0 mg, 99% yields). ^1^H NMR (400 MHz, DMSO-*d_6_*) δ 7.46 – 7.38 (m, 4H), 4.42 (t, *J* = 6.9 Hz, 1H), 3.21 – 3.08 (m, 2H), 2.55 (s, 3H), 2.37 (s, 3H), 1.59 (s, 3H). ESI-MS *m/z*: 401 [M+H]^+^.

**Synthesis of (*R*)-1-(4-((2-((*tert*-butoxycarbonyl)amino)phenyl)carbamoyl)phenyl)ethyl 2-((*R*)-4-(4-chlorophenyl)-2,3,9-trimethyl-6*H*-thieno[3,2-*f*][1,2,4]triazolo[4,3-*a*][1,4]diazepin-6-yl)acetate (17)**

To a solution of (-)-JQ1 carboxylic acid (**16**, 25.0 mg, 0.06 mmol), benzotriazol-1-yl-oxytripyrrolidinophosphonium hexafluorophosphate (0.1 g, 0.2 mmol) and *N*, *N*-diisopropylethylamine (31.1 mg, 0.2 mmol) in anhydrous dimethylformamide (2.0 mL) was added *tert*-butyl (*R*)-(2-(4-(1-hydroxyethyl)benzamido)phenyl)carbamate (**9**, 22.1 mg, 0.06 mmol) at 0 ℃. After addition, the reaction mixture was stirred at room temperature overnight. LC/MS analysis indicated the completed conversion. The resulting mixture was diluted with water (20.0 mL) and extracted with dichloromethane (20.0 mL * 3). The combined organic phases were washed with brine, dried over anhydrous sodium sulfate, filtered, concentrated under vacuum, absorbed onto silica gel, and purified via flash chromatography (ethyl acetate: hexanes = 1: 99 to 100% ethyl acetate) to afford a white form as (*R*)-1-(4-((2-((*tert*-butoxycarbonyl)amino)phenyl)carbamoyl)phenyl)ethyl 2-((*R*)-4-(4-chlorophenyl)-2,3,9-trimethyl-6*H*-thieno[3,2-*f*][1,2,4]triazolo[4,3-*a*][1,4]diazepin-6-yl)acetate (**17**, 3.1 mg, 7% yields). ^1^H NMR (400 MHz, CDCl_3_) δ 9.11 (s, 1H), 7.93 (d, *J* = 7.7 Hz, 2H), 7.73 (d, *J* = 7.8 Hz, 1H), 7.46 (d, *J* = 7.6 Hz, 2H), 7.41 – 7.26 (m, 5H), 7.22 – 7.10 (m, 2H), 6.81 (s, 1H), 6.01 – 5.92 (m, 1H), 4.58 (s, 1H), 3.77 – 3.57 (m, 2H), 2.65 (s, 3H), 2.38 (s, 3H), 1.65 (s, 3H), 1.61 (d, *J* = 6.6 Hz, 3H), 1.50 (s, 9H). ESI-MS *m/z*: 739 [M+H]^+^.

**Synthesis of (*R*)-1-(4-((2-aminophenyl)carbamoyl)phenyl)ethyl 2-((*R*)-4-(4-chlorophenyl)-2,3,9-trimethyl-6*H*-thieno[3,2-*f*][1,2,4]triazolo[4,3-*a*][1,4]diazepin-6-yl)acetate (009N1)**

To a stirred solution of (*R*)-1-(4-((2-((*tert*-butoxycarbonyl)amino)phenyl)carbamoyl)phenyl)ethyl 2-((*R*)-4-(4-chlorophenyl)-2,3,9-trimethyl-6*H*-thieno[3,2-*f*][1,2,4]triazolo[4,3-*a*][1,4]diazepin-6-yl)acetate (**17**, 6.0 mg, 0.008 mmol) in dichloromethane (2.0 mL) was added trifluoroacetic acid (0.5 mL). The reaction mixture was stirred at room temperature for 2 hours. LC/MS analysis indicated the completed conversion, and the resulting mixture was concentrated under vacuum to afford a light-yellow oil, which was diluted with 5.0 mL of dichloromethane. The mixture was basified via the addition of saturated sodium carbonate aqueous solution to pH = 10 and the resulting mixture was stirred at room temperature for 5 minutes. Then the mixture was extracted with dichloromethane (10.0 mL * 3). The combined organic phases were washed with brine, dried over anhydrous sodium sulfate, filtered, concentrated under vacuum, absorbed onto silica gel, and purified via flash chromatography (ethyl acetate: methanol = 1: 99 to 3: 97) to afford a beige foam as (*R*)-1-(4-((2-aminophenyl)carbamoyl)phenyl)ethyl 2-((*R*)-4-(4-chlorophenyl)-2,3,9-trimethyl-6*H*-thieno[3,2-*f*][1,2,4]triazolo[4,3-*a*][1,4]diazepin-6-yl)acetate (**009N1**, 4.1 mg, 80% yields). ^1^H NMR (400 MHz, DMSO-*d_6_*) δ 9.62 (s, 1H), 7.89 (d, *J* = 8.0 Hz, 2H), 7.48 (d, *J* = 8.1 Hz, 2H), 7.43 (d, *J* = 8.6 Hz, 2H), 7.27 (d, *J* = 8.2 Hz, 2H), 7.12 (d, *J* = 7.8 Hz, 1H), 6.93 (t, *J* = 7.6 Hz, 1H), 6.74 (d, *J* = 8.0 Hz, 1H), 6.56 (t, *J* = 7.5 Hz, 1H), 5.92 (q, *J* = 6.6 Hz, 1H), 4.85 (s, 2H), 4.48 (t, *J* = 7.3 Hz, 1H), 3.46 (dd, *J* = 7.2, 3.9 Hz, 2H), 2.56 (s, 3H), 2.37 (s, 3H), 1.56 (s, 3H), 1.49 (d, *J* = 6.5 Hz, 3H). ^13^C NMR (101 MHz, DMSO-*d_6_*) δ 170.31, 165.43, 163.91, 155.09, 150.51, 145.42, 143.71, 137.08, 135.86, 134.50, 132.87, 131.27, 130.68 (2C), 130.42, 129.97, 128.99 (2C), 128.49 (2C), 127.27, 127.06, 126.24 (2C), 123.75, 116.75, 116.62, 72.19, 53.99, 37.32, 22.63, 14.54, 13.22, 11.83. ESI-MS *m/z*: 639 [M+H]^+^. HPLC purity: 98.30%.

**5. Synthesis of (*S*)-1-(4-((2-aminophenyl)carbamoyl)phenyl)ethyl 2-((*R*)-4-(4-chlorophenyl)-2,3,9-trimethyl-6*H*-thieno[3,2-*f*][1,2,4]triazolo[4,3-*a*][1,4]diazepin-6-yl)acetate (009N2)**

**Synthesis of (*S*)-1-(4-((2-((*tert*-butoxycarbonyl)amino)phenyl)carbamoyl)phenyl)ethyl 2-((*R*)-4-(4-chlorophenyl)-2,3,9-trimethyl-6*H*-thieno[3,2-*f*][1,2,4]triazolo[4,3-*a*][1,4]diazepin-6-yl)acetate (18)**

To a solution of (-)-JQ1 carboxylic acid (**16**, 25.0 mg, 0.06 mmol), benzotriazol-1-yl-oxytripyrrolidinophosphonium hexafluorophosphate (0.1 g, 0.2 mmol) and *N*, *N*-diisopropylethylamine (31.1 mg, 0.2 mmol) in anhydrous dimethylformamide (2.0 mL) was added *tert*-butyl (*S*)-(2-(4-(1-hydroxyethyl)benzamido)phenyl)carbamate (**13**, 22.1 mg, 0.06 mmol) at 0 ℃. After addition, the reaction mixture was stirred at room temperature overnight. LC/MS analysis indicated the completed conversion. The resulting mixture was diluted with water (20.0 mL) and extracted with dichloromethane (20.0 mL * 3). The combined organic phases were washed with brine, dried over anhydrous sodium sulfate, filtered, concentrated under vacuum, absorbed onto silica gel, and purified via flash chromatography (ethyl acetate: hexanes = 1: 99 to 100% ethyl acetate) to afford a white form as (*S*)-1-(4-((2-((*tert*-butoxycarbonyl)amino)phenyl)carbamoyl)phenyl)ethyl 2-((*R*)-4-(4-chlorophenyl)-2,3,9-trimethyl-6*H*-thieno[3,2-*f*][1,2,4]triazolo[4,3-*a*][1,4]diazepin-6-yl)acetate (**18**, 3.3 mg, 7% yields). ^1^H NMR (400 MHz, CDCl_3_) δ 9.18 (s, 1H), 7.96 (d, *J* = 7.8 Hz, 2H), 7.82 (d, *J* = 7.9 Hz, 1H), 7.47 (d, *J* = 7.8 Hz, 2H), 7.30 – 7.24 (m, 5H), 7.23 – 7.11 (m, 2H), 6.77 (s, 1H), 5.97 (q, *J* = 6.6 Hz, 1H), 4.61 – 4.53 (m, 1H), 3.74 – 3.58 (m, 2H), 2.65 (s, 3H), 2.38 (s, 3H), 1.63 (s, 3H), 1.58 (d, *J* = 6.6 Hz, 3H), 1.48 (s, 9H). ESI-MS *m/z*: 739 [M+H]^+^.

**Synthesis of (*S*)-1-(4-((2-aminophenyl)carbamoyl)phenyl)ethyl 2-((*R*)-4-(4-chlorophenyl)-2,3,9-trimethyl-6*H*-thieno[3,2-*f*][1,2,4]triazolo[4,3-*a*][1,4]diazepin-6-yl)acetate (009N2)**

To a stirred solution of (*S*)-1-(4-((2-((*tert*-butoxycarbonyl)amino)phenyl)carbamoyl)phenyl)ethyl 2-((*R*)-4-(4-chlorophenyl)-2,3,9-trimethyl-6*H*-thieno[3,2-*f*][1,2,4]triazolo[4,3-*a*][1,4]diazepin-6-yl)acetate (**18**, 6.0 mg, 0.008 mmol) in dichloromethane (2.0 mL) was added trifluoroacetic acid (0.5 mL). The reaction mixture was stirred at room temperature for 2 hours. LC/MS analysis indicated the completed conversion, and the resulting mixture was concentrated under vacuum to afford a light-yellow oil, which was diluted with 5.0 mL of dichloromethane. The mixture was basified via the addition of saturated sodium carbonate aqueous solution to pH = 10 and the resulting mixture was stirred at room temperature for 5 minutes. Then the mixture was extracted with dichloromethane (20.0 mL * 3). The combined organic phases were washed with brine, dried over anhydrous sodium sulfate, filtered, concentrated under vacuum, absorbed onto silica gel, and purified via flash chromatography (ethyl acetate: methanol = 1: 99 to 3: 97) to afford a beige foam as (*S*)-1-(4-((2-aminophenyl)carbamoyl)phenyl)ethyl 2-((*R*)-4-(4-chlorophenyl)-2,3,9-trimethyl-6*H*-thieno[3,2-*f*][1,2,4]triazolo[4,3-*a*][1,4]diazepin-6-yl)acetate (**009N2**, 4.0 mg, 78% yields). ^1^H NMR (400 MHz, DMSO-*d_6_*) δ 9.65 (s, 1H), 7.95 (d, *J* = 8.1 Hz, 2H), 7.47 (d, *J* = 8.2 Hz, 2H), 7.43 (d, *J* = 8.5 Hz, 2H), 7.27 (d, *J* = 8.6 Hz, 2H), 7.14 (d, *J* = 6.5 Hz, 1H), 6.99 – 6.90 (m, 1H), 6.75 (d, *J* = 8.0 Hz, 1H), 6.56 (t, *J* = 7.6 Hz, 1H), 5.92 (q, *J* = 6.5 Hz, 1H), 4.87 (s, 2H), 4.46 (t, *J* = 7.3 Hz, 1H), 3.46 (d, *J* = 7.3 Hz, 2H), 2.56 (s, 3H), 2.37 (s, 3H), 1.56 (s, 3H), 1.49 (d, *J* = 6.6 Hz, 3H). ^13^C NMR (101 MHz, DMSO-*d_6_*) δ 170.41, 165.35, 163.78, 155.11, 150.44, 145.61, 143.74, 137.08, 135.90, 134.48, 132.91, 131.32, 130.63 (2C), 130.35, 129.95, 129.03 (2C), 128.47 (2C), 127.30, 127.09, 126.17 (2C), 123.75, 116.76, 116.65, 72.16, 54.08,37.14, 22.68, 14.57, 13.22, 11.81. ESI-MS *m/z*: 639 [M+H]^+^. HPLC purity: 99.01%.

**6. Synthesis of methyl 4-((*R*)-1-(2-((*S*)-4-(4-chlorophenyl)-2,3,9-trimethyl-6*H*-thieno[3,2-*f*][1,2,4]triazolo[4,3-*a*][1,4]diazepin-6-yl)acetoxy)ethyl)benzoate (009N3)**

To a stirred solution of (+)-JQ1 carboxylic acid (**5**, 40.0 mg, 0.1 mmol) and *N*, *N*-diisopropylethylamine (37.3 mg, 0.3 mmol) in anhydrous dimethylformamide (5.0 mL) was added benzotriazol-1-yl-oxytripyrrolidinophosphonium hexafluorophosphate (62.4 mg, 0.1 mmol) at 0 ℃. After stirring for 5 minutes, methyl (*R*)-4-(1-hydroxyethyl)benzoate (**7**, 19.8 mg, 0.1 mmol) was added and the resulting mixture was stirred at room temperature overnight. LC/MS analysis indicated the completed conversion. The reaction mixture was concentrated under vacuum, absorbed onto Celite and purified via C18 reversed flash chromatography (deionized water: methanol = 95: 5 to 100% methanol) to afford a white foam as methyl 4-((*R*)-1-(2-((*S*)-4-(4-chlorophenyl)-2,3,9-trimethyl-6*H*-thieno[3,2-*f*][1,2,4]triazolo[4,3-*a*][1,4]diazepin-6-yl)acetoxy)ethyl)benzoate (**009N3**, 32.0 mg, 56% yields). ^1^H NMR (400 MHz, DMSO-*d_6_*) δ 7.93 (d, *J* = 8.4 Hz, 2H), 7.50 (d, *J* = 8.1 Hz, 2H), 7.39 (d, *J* = 8.6 Hz, 2H), 7.25 (d, *J* = 8.6 Hz, 2H), 5.92 (q, *J* = 6.6 Hz, 1H), 4.45 (t, *J* = 7.3 Hz, 1H), 3.84 (s, 3H), 3.46 (d, *J* = 7.3 Hz, 2H), 2.56 (s, 3H), 2.36 (d, *J* = 0.9 Hz, 3H), 1.54 (d, *J* = 0.8 Hz, 3H), 1.48 (d, *J* = 6.6 Hz, 3H). ^13^C NMR (101 MHz, DMSO-*d_6_*) δ 170.28, 166.41, 163.62, 154.97, 150.32, 147.53, 136.98, 135.72, 132.80, 131.20, 130.51 (2C), 130.22, 129.80, 129.75 (2C), 129.33, 128.84 (2C), 126.55 (2C), 71.91, 53.95, 52.63, 36.99, 22.43, 14.44, 13.10, 11.70. ESI-MS *m/z*: 563 [M+H]^+^. HPLC purity: 96.86%.

**7. Synthesis of methyl 4-((*S*)-1-(2-((*S*)-4-(4-chlorophenyl)-2,3,9-trimethyl-6*H*-thieno[3,2-*f*][1,2,4]triazolo[4,3-*a*][1,4]diazepin-6-yl)acetoxy)ethyl)benzoate (009N4)**

To a stirred solution of (+)-JQ1 carboxylic acid (**5**, 40.0 mg, 0.1 mmol) and *N*, *N*-diisopropylethylamine (37.3 mg, 0.3 mmol) in anhydrous dimethylformamide (5.0 mL) was added benzotriazol-1-yl-oxytripyrrolidinophosphonium hexafluorophosphate (62.4 mg, 0.1 mmol) at 0 ℃. After stirring for 5 minutes, methyl (*S*)-4-(1-hydroxyethyl)benzoate (**11**, 19.8 mg, 0.1 mmol) was added and the resulting mixture was stirred at room temperature overnight. LC/MS analysis indicated the completed conversion. The reaction mixture was concentrated under vacuum, absorbed onto Celite and purified via C18 reversed flash chromatography (deionized water : methanol = 95 : 5 to 100% methanol) to afford a white foam as methyl 4-((*S*)-1-(2-((*S*)-4-(4-chlorophenyl)-2,3,9-trimethyl-6*H*-thieno[3,2-*f*][1,2,4]triazolo[4,3-*a*][1,4]diazepin-6-yl)acetoxy)ethyl)benzoate (**009N4**, 27.0 mg, 48% yields). ^1^H NMR (400 MHz, DMSO-*d_6_*) δ 7.86 (d, *J* = 8.3 Hz, 2H), 7.50 (d, *J* = 8.1 Hz, 2H), 7.40 (d, *J* = 8.6 Hz, 2H), 7.24 (d, *J* = 8.6 Hz, 2H), 5.93 (q, *J* = 6.6 Hz, 1H), 4.49 – 4.44 (m, 1H), 3.80 (s, 3H), 3.49 – 3.41 (m, 2H), 2.55 (s, 3H), 2.41 – 2.35 (m, 3H), 1.56 (s, 3H), 1.48 (d, *J* = 6.6 Hz, 3H). ^13^C NMR (101 MHz, DMSO-*d_6_*) δ 170.24, 166.42, 163.82, 155.07, 150.50, 147.39, 136.97, 135.83, 132.89, 131.26, 130.67 (2C), 130.38, 129.92, 129.87 (2C), 129.48, 128.92 (2C), 126.72 (2C), 72.01, 53.95, 52.68, 37.30, 22.44, 14.50, 13.20, 11.82. ESI-MS *m/z*: 563 [M+H]^+^. HPLC purity: 99.38%.
